# An Improved Design of the MultiCal On-Site Calibration Device for Industrial Robots

**DOI:** 10.3390/s23125717

**Published:** 2023-06-19

**Authors:** Ziwei Wan, Chunlin Zhou, Zhaohui Lin, Huapeng Yan, Weixi Tang, Zheng Wang, Jun Wu

**Affiliations:** 1 College of Control Science and Engineering, Zhejiang University, Hangzhou 310063, China; wanzw@zju.edu.cn (Z.W.);; 2 Laboratory of Medical Robots, Huzhou Institute of Zhejiang University, Huzhou 313098, China; 3 School of Mechanical Engineering, Zhejiang University, Hangzhou 310030, China; 4 School of Automation Engineering, University of Electronic Science and Technology of China, Chengdu 611731, China; 5 School of Engineering, Huzhou University, Huzhou 313000, China

**Keywords:** calibration device, kinematic calibration, on-site calibration, industrial robot, accuracy measurement

## Abstract

MultiCal is an affordable, high-precision measuring device designed for the on-site calibration of industrial robots. Its design features a long measuring rod with a spherical tip that is attached to the robot. By restricting the rod’s tip to multiple fixed points under different rod orientations, the relative positions of these points are accurately measured beforehand. A common issue with MultiCal is the gravitational deformation of the long measuring rod, which introduces measurement errors into the system. This problem becomes especially serious when calibrating large robots, as the length of the measuring rod needs to be increased to enable the robot to move in a sufficient space. To address this issue, we propose two improvements in this paper. Firstly, we suggest the use of a new design of the measuring rod that is lightweight yet has high rigidity. Secondly, we propose a deformation compensation algorithm. Experimental results have shown that the new measuring rod improves calibration accuracy from 20% to 39%, while using the deformation compensation algorithm, the accuracy increases from 6% to 16%. In the best configuration, the calibration accuracy is similar to that of a measuring arm with a laser scanner, producing an average positioning error of 0.274 mm and a maximum positioning error of 0.838 mm. The improved design is cost-affordable, robust, and has sufficient accuracy, making MultiCal a more reliable tool for industrial robot calibration.

## 1. Introduction

**Figure 1:**
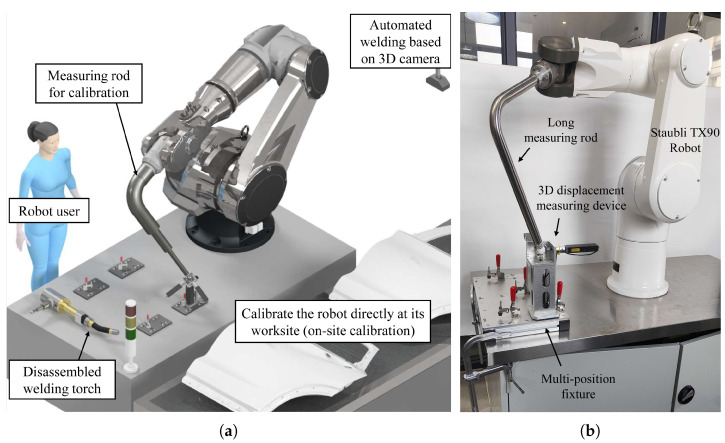
(**a**) On-site kinematic calibration for an industrial robot and (**b**) MultiCal [6].

Robot calibration involves measuring a robot’s end effector at different joint angles using high-precision measuring equipment to determine accurate kinematic parameters. Currently, calibration is typically performed after a robot’s manufacturing, known as in-house calibration. The advantages of in-house calibration include: First, the calibrated parameters can be embedded directly into the robot controller [1]. Second, purchasing expensive measuring equipment, such as laser trackers [2], optical CMM [3], and even CMM [4], is cost-effective due to their high use frequency. However, the accuracy of robots degrades as they are continuously used, so it is necessary to monitor the robots’ accuracy online and conduct on-site recalibration (Figure 1a) when the accuracy is severely degraded [5]. On-site calibration is especially important for robots with off-line programming and visual navigation, as the absolute accuracy of a robot, rather than its higher repeatability, ensures that the motion instructions can be directly used for real tasks. Although some manufacturers provide on-site calibration services, most still have to rent expensive laser trackers from a local provider of metrology services [1]. This has spurred many researchers to develop various portable and affordable measuring devices for on-site robot calibration.

When it comes to calibration devices, the requirements for on-site equipment differ from those for in-house calibration. Ideally, on-site equipment should have high calibration accuracy, low cost, good robustness, portability, good environmental adaptability (able to be used in small robot cells), and versatility (suitable for different-sized robots), while also being able to monitor robot accuracy online. Note that on-site calibration equipment does not require full automation or extremely high time efficiency since calibration frequency is not high and manual interventions must be involved (to arrange the measuring devices). Additionally, the device must measure the robot in a large portion of its motion space to ensure high calibration accuracy. By keeping these requirements in mind, real industrial applications can choose the best on-site calibration device to fit their needs.

MultiCal [6] (Figure 1b) is such a measuring device designed for the on-site calibration of industrial robots. Its design features a long measuring rod with a spherical tip that is attached to the robot. The calibration procedure of MultiCal involves aligning the rod’s tip to multiple fixed points under different rod orientations, the relative positions of these points are accurately measured beforehand. However, for large robot calibration, the measuring rod of MultiCal has to be elongated and may enlarge the gravitational deformation of the rod, thus increasing measurement errors in the system. To address this issue, we propose two improvements in this paper. Firstly, we suggest a new design of the measuring rod that is lightweight and has high rigidity. Secondly, a deformation compensation algorithm is proposed to reduce the error. The work is mostly based on the assumptions and experiment described in our previously published paper on MultiCal [6]. Details of the improvements are discussed in the following sections.

The remainder of this paper is organized as follows. Section 2 reviews and analyses related works. In Section 3, a new design scheme and calibration method of the MultiCal is discussed. Section 4 analyses the deformation of the measuring rod. The experimental setup and results are proposed in Section 5. Comparisons of different measuring devices and methods are also discussed in this section. Finally, the paper is concluded in Section 6.

## 2. Related Works

Current calibration devices can be divided into two categories: open- and closed-loop, each utilizing different calibration methods. Open-loop devices require markers to be installed on the robot’s end effector, and measurements are performed using devices such as laser trackers [2], optical CMMs [3], CMMs [4], binocular vision [7], and wire draw encoders [8]. This method allows for almost unlimited robot poses, and measurements can be automatically collected over a large workspace. Therefore, these devices (especially laser trackers) are commonly used for in-house calibration. However, open-loop devices may be obstructed in small robot cells and are often very expensive (>50 K USD).

Closed-loop devices rely on probes [9], displacement sensors [10], optical sensors [11], precision balls [12], and standard blocks [13]. A typical closed-loop method is to restrict the robot’s tool centre point (TCP) to a fixed point and then change the orientation of the end effector. These measuring devices inherently have the advantages of good environmental adaptability and low cost. However, their major limitation is that the robot must be restricted to a certain pose to collect the measurements, leading to degraded calibration accuracy and collision risks.

To overcome the limitations of open-loop calibration devices and make them more suitable for on-site calibration, closed-loop devices based on multi-point constraints have been proposed. Two examples of such devices are TriCal [14] and MultiCal [6]. These devices use a similar measurement process, in which the robot automatically aligns its TCP with multiple fixed points, based on the feedback of three displacement sensors. By using multi-point constraints, these devices achieve a stronger constraint and a larger measurement space, resulting in good calibration accuracy and robustness.

Compared to TriCal, MultiCal uses a precision ball instead of a heavier 3D displacement measuring device at the end of its measuring rod. This design reduces the rod’s gravitational deformation while allowing for a longer measuring rod, resulting in increased motion space and improved calibration accuracy for the robot. Although the measurement process of MultiCal is more complex than TriCal, due to the need for manual switching of the measuring device to different clamping positions, it is less prone to collisions and more robust. This is because its displacement sensors have a larger measurement range of 30 mm, and the multi-position fixture has no vulnerable components critical to system accuracy, such as the extension stems. MultiCal is therefore well-suited for on-site calibration devices that are not frequently used and typically operated by non-professionals. However, our previous work [6] showed that even though MultiCal minimized the load on the measuring rod as much as possible, its lengthened stainless-steel measuring rod still suffered from gravitational deformation, which could result in decreased calibration accuracy.

Deformation of structural components is a common problem in many measuring or machining devices, especially those involving slender structural components, which results in a decrease in measurement or machining accuracy. To address this issue, the conventional method is to estimate and compensate for deformation errors using elastic modelling [15], finite element analysis (FEA) [16], experimental measurement [17], or neural network estimation [18]. As the gravitational deformation of the measuring rod is a relatively simple elastic deformation problem, this article adopts the first three methods based on considerations of robustness and accuracy.

## 3. Improvement of the Calibration Devices and Methods

This section first describes the detailed design scheme of MultiCal. Then the robot kinematics model, non-kinematic parameters, calibration algorithm, and compensation algorithm for the rod’s gravitational deformation used during calibration are presented.

### 3.1. Design of MultiCal and the Measuring Rod

**Figure 2:**
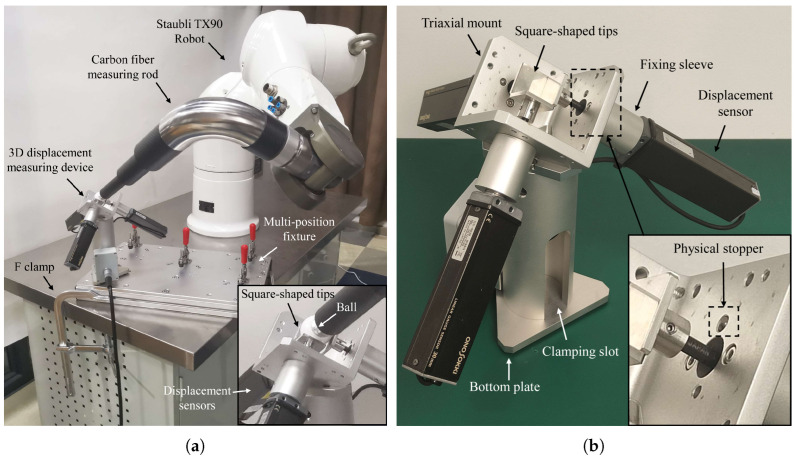
(**a**) MultiCal with the improved measuring rod and (**b**) a redesigned 3D displacement measuring device.

The improved design of MultiCal is illustrated in Figure 2a. The device comprises three major components: (1) a long carbon fibre measuring rod with a spherical tip made up of a precision ceramic ball installed at the end of the robot, (2) a redesigned 3D displacement measuring device with a μm-level accuracy, and (3) a fixture providing multiple clamping positions that are fixed with respect to the robot’s base. MultiCal adopts the automatic alignment of the TCP (the spherical tip’s centre) with a fixed point based on the feedback from three displacement sensors. Note that the measuring rod does not need high dimensional accuracy but needs high rigidity and lightweight.

**Figure 3:**
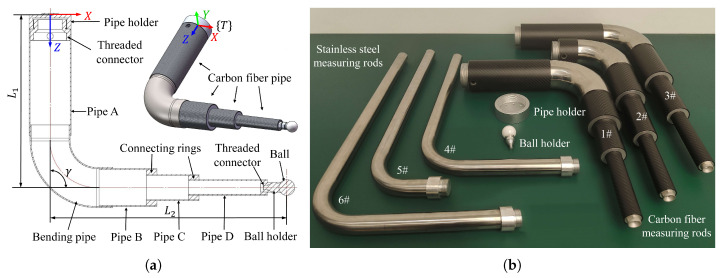
(**a**) Design of carbon fibre measuring rod and (**b**) different types of rods.

The measuring rod is improved by using a new structure and carbon fibre pipes, as depicted in Figure 3a. Two high-strength aluminium rings connect different carbon fibre pipes, and a stainless-steel bending pipe is used at the bend. All parts of the rod are bonded with high-strength epoxy resin adhesive. The pipe and ball holders are installed at the start and end of the measuring rod using threaded connectors, used for rapid installation and removal with the robot flange and precision ball. Three different configurations of the carbon fibre rods (Figure 3b, see Section S1 of the Supplementary Materials for the detailed design parameters) are developed to replace the previously-used stainless-steel measuring rods in [6].

(1) $$\chi_{T}=\left[\begin{array}{c} x_{tool}\\ y_{tool}\\ z_{tool} \end{array}\right]=\left[\begin{array}{c} L_{2}\sin\left(\gamma \right)\\ 0\\ L_{1}-L_{2}\cos\left(\gamma \right) \end{array}\right]$$

The measuring rod is mounted as an end-effector on the robot, and the position $\chi_{T}$ of the precision ball’s centre with respect to the tool frame {T} (tool parameters) can be calculated by Equation (1) as nominal values. As shown in Figure 2b, the 3D displacement measuring device measures the displacement of the centre of the measuring rod’s spherical tip. Three displacement sensors are installed orthogonally on an aluminium triaxial mount, ensuring that the sensors’ measuring axes are perpendicular to each other.

The measurement process involves making contact between the precision ball and the square-shaped tips of the sensors. The sensors are zeroed by pushing each square-shaped tip into contact with a physical stopper (a tungsten steel ball) set on the triaxial mount. In contrast to [6], all three measuring axes of the sensors are inclined upwards at the same angle (approximately 54.7°) relative to the plumb axis. This design provides a larger measuring space in the vertical upward direction, enabling the robot to move more freely.

Using the feedback from the displacement sensors, the robot can adjust its TCP to a fixed point in *k* different orientations and ensure that the measured values of all sensors are almost exactly half of their ranges. The corresponding *k* sets of joint angles $q_{i}\;\left(i=1,2,\ldots ,k\right)$ and the small displacement deviation $x_{i}\;\left(i=1,2,\ldots ,k\right)$ are then recorded. This process is designed to automatically [14] or semi-automatically [6] achieve a point constraint with minimal contact force, resulting in minimal deformation, fast efficiency, and high measurement accuracy. The constrained point is marked as the virtual datum point.

**Figure 4:**
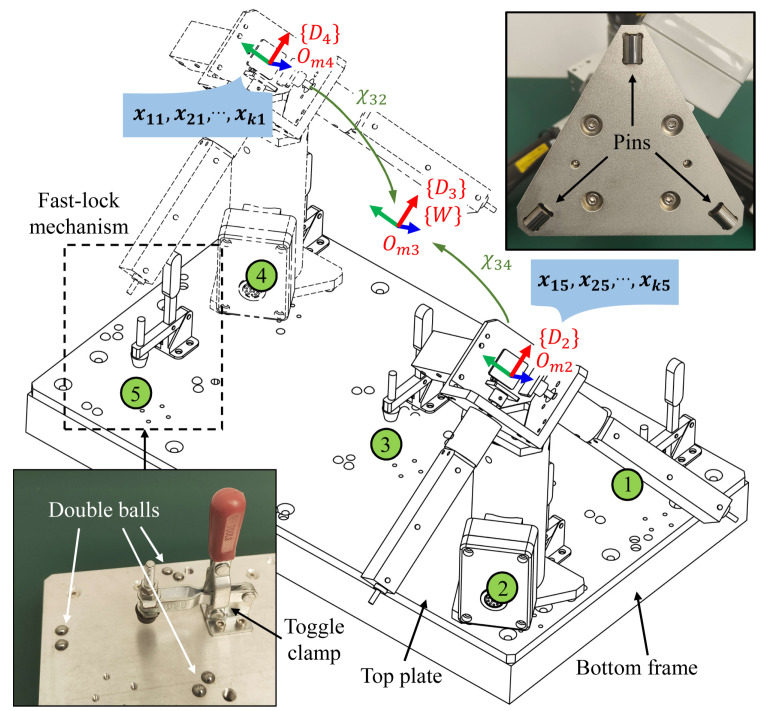
Use of a multi-position fixture to provide five clamping positions.

The MultiCal system also includes a fixture with multiple clamping positions fixed relative to the robot’s base, such as the multi-position fixture shown in Figure 4. This fixture is designed to be compact and lightweight, making it easy to transport and embed into the robot’s cells. Equipped with multiple fast-lock mechanisms, comprising a toggle clamp and three sets of point positioning components made of tungsten steel. These components, consisting of a single pin and two balls spaced at a certain distance, ensure assembly accuracy.

The fast-lock mechanisms allow users to quickly mount the 3D measuring device on different clamping positions (less than 15 s). Repeating the above measurement process obtains the corresponding measurement data $x_{ij}$ and $q_{ij}$ (i=1,2,…,k; j=1,2,…,5) (assuming that five clamping positions are used). The displacement deviation $x_{ij}$ is regarded as the coordinate of the *i*th point measured on the *j*th clamping position with respect to the device’s frame $\left\{D_{j}\right\}$ (j=1,2,…,5). A hexagon measuring arm is used to measure the relative positions between different the device’s frame (see Section S2 of the Supplementary Materials for the specific method), which can convert the coordinate points in different frames to a single frame ($\left\{D_{3}\right\}$ chosen in this paper), marked as the world frame {W}.

Specifically, the relative poses between $\left\{D_{3}\right\}$ and the other device frames are recorded as $\chi_{3j}$ (j=1,2,4,5), which are 6D pose vectors comprising XYZ coordinates and Euler angles. The equation for the coordinate conversion is:

(2) $$x_{ij}^{meas}=T\left(\chi_{3j}\right)x_{ij}$$

where $T(\chi_{3j})$ is the function transforming the 6D pose vector into a transformation matrix, and $x_{ij}^{meas}$ is the measured coordinates of the measurement point with respect to the world frame {W}. The above process essentially achieves multi-point constraints, thereby providing stronger constraints and making the kinematics parameter identification more robust. For a large robot, another solution involves permanently fixing multiple single-position fixtures near the robot base (Figure 1a) and then using an external measurement device (such as a measuring arm or laser tracker) to measure $\chi_{3j}$ (j=1,2,4,5) before the long-term use of these fixtures.

### 3.2. Calibration Method

**Figure 5:**
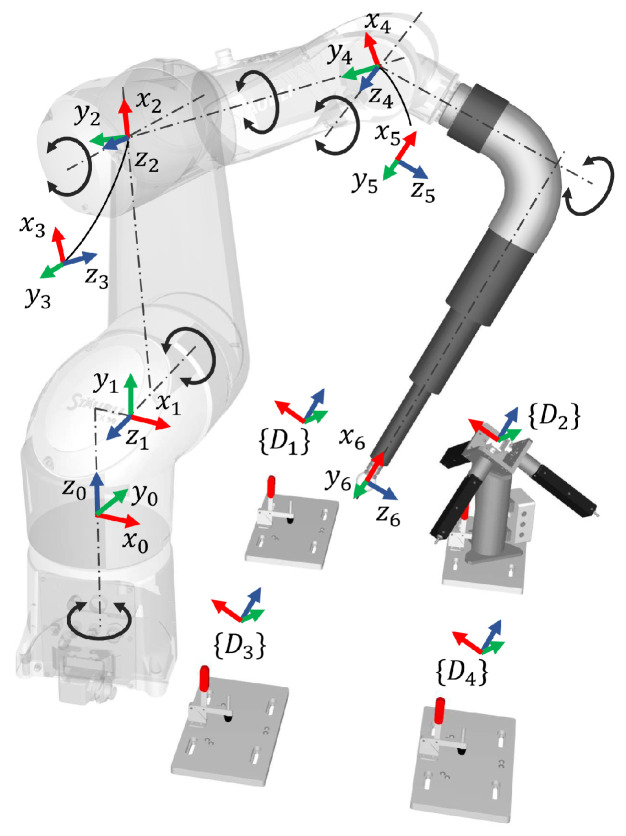
Modified DH (MDH) model of the Staubli TX90 robot.

This section presents the robot kinematic model, the parameters that need to be identified, and the calibration algorithm. In this paper, we have chosen the Staubli TX90 robot (Figure 5) as a representative for validating the effectiveness of our method. This model features a classic 6-DoF configuration and can effectively represent a significant portion of medium-sized robots. For robots of different sizes and configurations, the measuring rod’s geometric dimensions can be reselected and optimized following the approach outlined in this paper and [6].

To begin the calibration process, we first establish a kinematic model and determine the parameters that require identification. In our previous work [6], we compared different kinematic modelling methods, including Denavit–Hartenberg (DH), modified DH (MDH) [19], product of exponential (POE) [20], and finite and instantaneous screw (FIS) [21]. After considering implementation and promotion difficulty, we ultimately selected the MDH method.

As shown in Table 1, we define the centre of the precision ball as the origin of the last frame (TCP), and add a rotation angle β around the *y*-axis to the adjacent parallel joints (link 1–2) to eliminate the singularity. Additionally, $d_{3}$ must be zero. Furthermore, both $\theta_{1}$ and $d_{1}$ are coupled with the 6D base parameter $\chi_{B}$ from the robot base frame {0} to the world frame {W}, and are thus not included in parameter identification. The compliance in the gearboxes of all joints, except the first, is modelled as a linear torsional spring [14], as no torque is applied to the first joint axis when the robot is stationary. We combine these kinematic and non-kinematic parameters into the parameter vector ρ that we aim to identify.

(3) $$\rho =\left[\delta \theta^{T},\delta d^{T},\delta a^{T},\delta \alpha^{T},\delta \beta_{2},\chi_{B},\chi_{T},c^{T}\right]$$

where δθ, δd, δa, δα, and $\delta \beta_{2}$ are the errors of the MDH parameters, $\chi_{B}$ is the vector of robot base parameters, $\chi_{T}$ is the vector of tool parameters, c is the vector of the compliance coefficient of the joint gearboxes, and $\tau_{s}$ (s=2,3,…,6) is the torque applied on the five joints, which is calculated using the iterative Newton–Euler algorithm [14]. Among them, $\chi_{B}$ is a 6D vector, including XYZ coordinates and Euler angles, as presented in Equation (4).

(4) $$\chi_{B}=\left[x_{0}^{W},y_{0}^{W},z_{0}^{W},\alpha_{0}^{W},\beta_{0}^{W},\gamma_{0}^{W}\right]$$

It is worth noting that the error associated with the tool parameters, ${\delta x}_{tool}$ and ${\delta z}_{tool}$, and the tool angler error, ${\delta \theta }_{6}$, are relatively larger due to the low dimensional tolerance of the measuring rods. The transformation matrix $T_{T}^{W}$ between the tool frame {T} and the world frame {W} can be calculated based on the robot forward kinematics equation,

(5) $$T_{T}^{W}\left(\rho ,q,\tau \right)=T_{0}^{W}T_{1}^{0}T_{2}^{1}T_{3}^{2}T_{4}^{3}T_{5}^{4}T_{6}^{5}T_{T}^{6}$$

where $T_{0}^{W}$, $T_{n}^{n-1}$(n=1,2,⋯,6), and $T_{T}^{6}$ are the transformation matrix between the robot base frame and the world frame, adjacent robot link frames, and the robot flange frame and tool frame, respectively. Among them, the values of $T_{0}^{W}$ and $T_{T}^{6}$ are determined by the robot base parameters $\chi_{B}$ and tool parameters $\chi_{T}$, respectively. Based on Equation (5), the TCP’s nominal coordinates (the position vector of $T_{T}^{W}$) with respect to the world frame {W} can be obtained.

(6) $$x_{ij}^{nominal}=\left[\begin{array}{c} x_{T}^{W}\\ y_{T}^{W}\\ z_{T}^{W} \end{array}\right]=f\left(\rho ,q_{ij},\tau_{ij}\right)$$

where $x_{ij}^{nominal}$ is the nominal coordinate of the *i*th point (i=1,2,…,k) measured on the *j*th clamping position (j=1,2,…,5), and $\tau_{ij}$ is the vector of torques applied on the robot joints when measuring this point. Then, a linear error model is established based on the difference between the measured coordinates $x_{ij}^{meas}$ and the nominal coordinates $x_{ij}^{nominal}$, and the error vector $\Delta \overset{˘}{\rho }$ can be solved by the least square method.

(7) $$\Delta x=\left[\begin{array}{c} x_{11}^{meas}-x_{11}^{nominal}\\ x_{12}^{meas}-x_{12}^{nominal}\\ \vdots \\ x_{k5}^{meas}-x_{k5}^{nominal} \end{array}\right]=J\Delta \overset{˘}{\rho }$$

(8) $$\Delta \overset{˘}{\rho }=J^{+}\Delta x={\left(J^{T}J\right)}^{-1}J^{T}\Delta x$$

Since the above linear error model still has errors, $\overset{˘}{\rho }$ can be optimized iteratively using the Levenberg–Marquardt (LM) algorithm, commonly used in robot kinematics [22] and has strong robustness. Essentially, the identification problem becomes an optimization problem.

(9) $$\overset{˘}{\rho }=\arg\min\sum_{i=1}^{k}\sum_{j=1}^{5}{∥x_{ij}^{meas}-f\left(\rho ,q_{ij},\tau_{ij}\right)∥}^{2}$$

**Figure 6:**
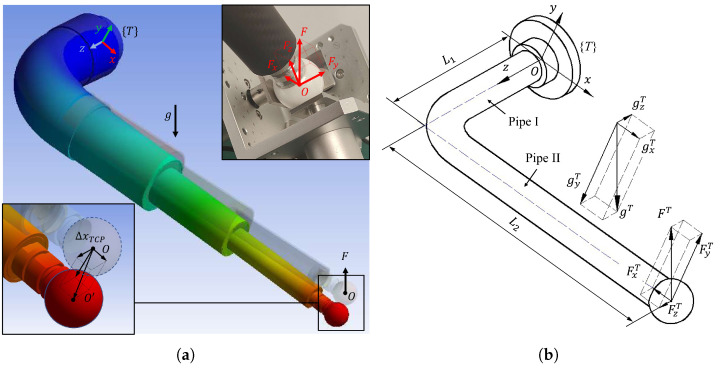
(**a**) Measuring the rod’s deformation caused by gravity and contact forces; and (**b**) force decomposition.

Figure 6a illustrates the TCP offset ($\Delta x_{TCP}$) caused by rod gravity and the reaction force F from the measuring sensors. This reaction force F comprises the contact forces of the three displacement sensor tips ($F_{x},F_{y},F_{z}$), which are accurately measured using a force-measuring device. As the axes of the three sensors are parallel to the XYZ axis of the device frame $\left\{D_{j}\right\}$ (j=1,2,…,5), the contact force values of the three tips when the ball’s centre coincides with the virtual datum point are directly used as the vector value of $F^{D}$ with respect to the frame $\left\{D_{j}\right\}$. Since the TCP is aligned with the same point fixed with respect to the device each time, the vector value of $F^{D}$ (determined by the compression of the springs) is considered constant in each measurement.

The TCP offset vector of the measuring rod when measuring the *i*th point on the *j*th clamping position is marked as $\Delta x_{TCP}^{ij}$, which can be decomposed into the XYZ direction of the tool frame {T}.

(10) $$\Delta x_{TCP}^{ij}=\left[\begin{array}{c} x_{TCP}^{T}\\ y_{TCP}^{T}\\ z_{TCP}^{T} \end{array}\right]$$

Then the measured coordinates of the TCP can be compensated for to obtain the corrected coordinates $x_{ij}^{\prime \;meas}$ of this point.

(11) $$x_{ij}^{\prime \;meas}=x_{ij}^{meas}-R_{T}^{W}\Delta x_{TCP}^{ij}$$

where $R_{T}^{W}$ is the rotation matrix between the world frame {W} and the tool frame {T}. We use $x_{i}^{\prime \;meas}$ to replace $x_{i}^{meas}$ in Equation (9), and the calibration after compensation can be conducted.

## 4. Study of the Measuring Rod’s Deformation

In this section, we discuss the elastic deformation model of the measuring rod caused by gravity and the contact force, which can degrade the measurement accuracy and require estimation and compensation. We then present how the compliance parameters associated with this model are obtained through a finite element analysis (FEA) and a measurement experiment.

### 4.1. Elastic Deformation Model

The simplified measuring rod model and the external force decomposition is depicted in Figure 6b. The measuring force vector $F^{D}$ of the measuring device mounted on different clamping positions can be converted into the world frame {W}:

(12) $$F_{j}^{W}=R\left(\chi_{3j}\right)F^{D}$$

where $F_{j}^{W}$ is the measuring force vector (with respect to the world frame {W}) of the 3D measuring device mounted on the *j*th clamping position, $R(\chi_{3j})$ is the function transforming the Euler angles of the 6D pose vector $\chi_{3j}$ into a rotation matrix. After this, $F_{j}^{W}$ is converted to the tool frame {T}:

(13) $$F_{ij}^{T}=R_{6}^{T}R_{0}^{6}R_{W}^{0}F_{j}^{W}$$

where $F_{ij}^{T}$ is the measuring force vector with respect to the tool frame {T} when measuring the *i*th point on the *j*th clamping position, $R_{6}^{T}$ is the rotation matrix of the robot flange frame {6} and the tool frame {T} (determined by the tool parameters $\chi_{T}$), $R_{0}^{6}$ is the rotation matrix between the robot’s base frame {0} and the flange frame {6} when measuring this point (determined by the robot kinematic model and joint angles $q_{ij}$), and $R_{W}^{0}$ is the rotation matrix between the robot’s base frame {0} and the world frame {W} (determined by the base parameters $\chi_{B}$). The robot first needs to be calibrated without considering the rod’s deformation to determine the “rough values” of the MDH parameters. This process is also called “rough calibration”. Experiments have proven that these MDH parameters are sufficiently accurate to estimate the measuring rod’s deformation.

The gravitational acceleration vector g can be also converted into the tool frame {T}:

(14) $$g_{ij}^{T}=R_{6}^{T}R_{0}^{6}g^{0}=R_{6}^{T}R_{0}^{6}g$$

where $g_{ij}^{T}$, $g^{0}$, and g are the gravitational acceleration vectors with respect to the tool frame {T}, robot’s base frame {0}, and global frame when measuring the *i*th point on the *j*th clamping position, respectively. Then, $F_{ij}^{T}$ and $g_{ij}^{T}$ are decomposed into the XYZ direction of the tool frame {T} (Figure 6b). According to the principle of linear superposition in material mechanics, we analyse the deformation of pipes I and II caused by the XYZ components of $F_{ij}^{T}$ and $g_{ij}^{T}$, respectively, and then add them up. Then, the offset vector $\Delta x_{ij}^{F}$ of the TCP caused by the measuring force of the 3D measuring device mounted on the *i*th clamping position can be calculated based on Equation (15) (see Section S3 of Supplementary Materials for the detailed derivation process).

(15) $$\Delta x_{ij}^{F}=\left[\begin{array}{c} \Delta x_{xij}^{F}\\ \Delta x_{yij}^{F}\\ \Delta x_{zij}^{F} \end{array}\right]=\left[\begin{array}{ccc} \frac{L_{1}^{3}}{3EI_{Z}} & 0 & -\frac{L_{1}^{2}L_{2}}{2EI_{Z}}\\ 0 & \frac{L_{1}^{3}+L_{2}^{3}}{3EI_{Z}}+\frac{L_{1}L_{2}^{2}}{GI_{P}} & 0\\ -\frac{L_{1}^{2}L_{2}}{2EI_{Z}} & 0 & \frac{L_{2}^{3}}{3EI_{Z}}+\frac{L_{1}L_{2}^{2}}{EI_{Z}} \end{array}\right]\left[\begin{array}{c} F_{xij}^{T}\\ F_{yij}^{T}\\ F_{zij}^{T} \end{array}\right]$$

where *E*, *G*, $I_{z}$ and $I_{z}$ are the elastic modulus, shear modulus, moment of inertia, and polar moment of inertia of the hollow pipes, respectively. Each element of the matrix in Equation (15) can be defined as a compliance coefficient $c_{mn}^{F}$ (m=1,2,3; n=1,2,3) of the measuring rod in the corresponding direction:

(16) $$\Delta x_{ij}^{F}=\left[\begin{array}{ccc} c_{11}^{F} & 0 & c_{13}^{F}\\ 0 & c_{22}^{F} & 0\\ c_{31}^{F} & 0 & c_{33}^{F} \end{array}\right]F_{ij}^{T}=C^{F}F_{ij}^{T}$$

where $C^{F}$ is the compliance matrix for the measuring force, an inherent property of the measuring rod. Instead of stiffness, compliance (the reciprocal of stiffness) is used to make the formula more concise. Note that $F_{xij}^{T}$ and $F_{zij}^{T}$ will only cause TCP offset in the *X* and *Z* directions, while $F_{yij}^{T}$ will only cause TCP offset in the *Y* direction.

Similarly, the TCP offset vector $\Delta x_{ij}^{g}$ (with respect to the tool frame {T}) caused by gravity is calculated based on Equation (17).

(17) $$\Delta x_{ij}^{g}=\left[\begin{array}{c} \Delta x_{xij}^{g}\\ \Delta x_{yij}^{g}\\ \Delta x_{zij}^{g} \end{array}\right]=\left[\begin{array}{ccc} \frac{L_{1}^{3}L_{2}}{3EI_{Z}}+\frac{L_{1}^{4}}{8EI_{Z}} & 0 & -\frac{L_{1}^{2}L_{2}^{2}}{4EI_{Z}}\\ 0 & \frac{L_{2}^{4}}{8EI_{Z}}+\frac{L_{1}^{3}L_{2}}{3EI_{Z}}+\frac{L_{1}^{4}}{8EI_{Z}}+\frac{L_{1}L_{2}^{3}}{2GI_{P}} & 0\\ -\frac{L_{1}^{3}L_{2}}{6EI_{Z}}-\frac{L_{1}^{2}L_{2}^{2}}{2EI_{Z}} & 0 & \frac{L_{2}^{4}}{8EI_{Z}}+\frac{L_{1}L_{2}^{3}}{2EI_{Z}} \end{array}\right]\left[\begin{array}{c} q_{xij}^{T}\\ q_{yij}^{T}\\ q_{zij}^{T} \end{array}\right]$$

where $q^{T}={\left[q_{xij}^{T},q_{yij}^{T},q_{zij}^{T}\right]}^{T}$ is the uniform load exerted by gravitational acceleration on the hollow pipe, the section shape of the hollow pipe, and the material density. We extract the gravitational acceleration vector $g_{ij}^{T}$ in $q_{ij}^{T}$ by:

(18) $$\Delta x_{ij}^{g}=\left[\begin{array}{ccc} c_{11}^{g} & 0 & c_{13}^{g}\\ 0 & c_{22}^{g} & 0\\ c_{31}^{g} & 0 & c_{33}^{g} \end{array}\right]g_{ij}^{T}=C^{g}g_{ij}^{T}$$

where $C^{g}$ is the compliance coefficient matrix for gravity, another inherent property of the measuring rod. In summary, we only need to determine the compliance coefficients $c_{mn}^{F}$ and $c_{mn}^{g}$ (m=1,2,3; n=1,2,3) in $C^{F}$ and $C^{g}$. Then the final overall TCP offset vector $\Delta x_{TCP}^{ij}$ can be estimated and compensated for using Equations (19) and (11).

(19) $$\Delta x_{TCP}^{ij}=\Delta x_{ij}^{g}+\Delta x_{ij}^{F}=C^{g}g_{ij}^{T}+C^{F}F_{ij}^{T}$$

### 4.2. Determination of Rod Compliance Coefficients

**Figure 7:**
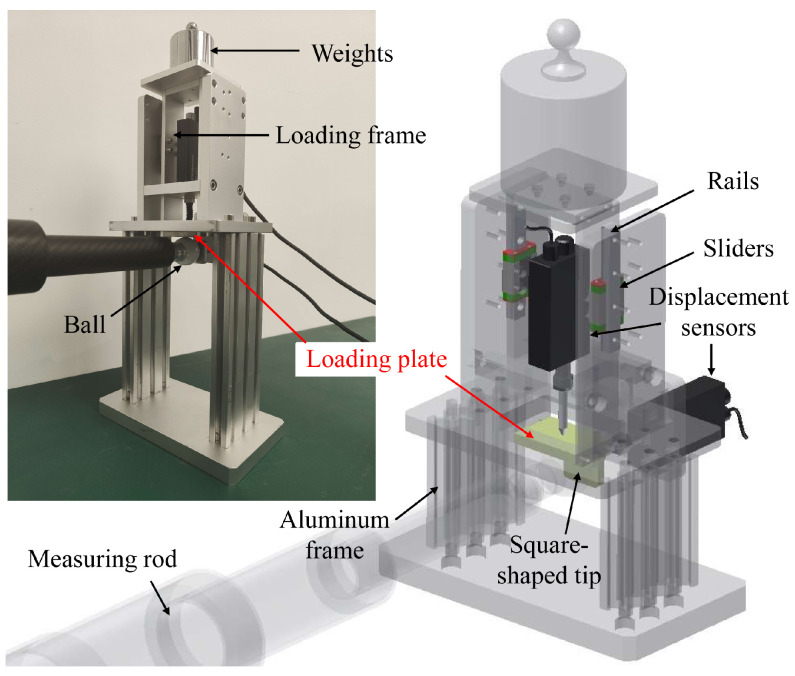
Close-up of the stiffness-measuring device.

To estimate the rod deformation error using the model derived above, we need to determine the compliance coefficients of the measuring rod in the XYZ directions. To achieve this, we conducted both an FEA (Figure 6a) and a real measurement experiment to obtain these parameters. Firstly, we imported the 3D model of the measuring rod, built in SolidWorks, into ANSYS to perform a static deformation analysis. This allowed us to obtained the maximum directional deformation values of the spherical tip in the XYZ directions, approximating the TCP offset values. These values were used to calculate the corresponding compliance coefficients. Secondly, a two-dimensional stiffness-measuring device was developed to determine the stiffness through measurements (Figure 7, see Section S4 of the Supplementary Materials for detailed design and measurement results). By putting different weights on the loading frame, we can apply different loads to the measuring rod and measure its vertical and horizontal TCP offset under the load. The TCP offset measurement method satisfies the Abbe measuring principle [23] and eliminates errors caused by rotation of the loading plate.

**Figure 8:**
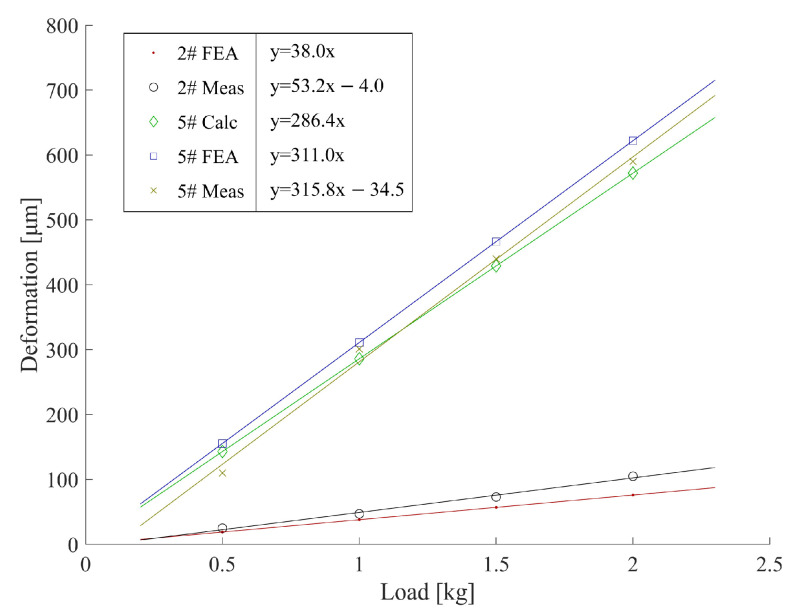
Load-deformation result of the 2# (carbon fibre) and 5# (stainless-steel) measuring rods using the theoretical calculation (Calc), FEA, and real measurement (Meas) methods.

Moreover, a correction factor *r* is defined to characterize the difference between the FEA-simulated stiffness and the measured stiffness:

(20) $$r_{mn}=\frac{c_{mn}^{F}}{c_{mn}^{\prime \;F}}\;\left(m=1,2,3;n=1,2,3\right)$$

where $c_{mn}^{F}$ is the slope of the fitting lines of the actual measured load-deformation result (Figure 8), and $c_{mn}^{\prime \;F}$ is the compliance coefficient obtained by the FEA result. The correction factor $r_{mn}$ of a measuring rod in different directions calculated by Equation (20) is usually different. In the direction with a small deformation (high stiffness), the correction factor $r_{mn}$ is more susceptible to the measurement error. Therefore, we assume the correction factor $r_{mn}$ of all directions is a fixed value since the measuring rod has a certain symmetry. The two directions with the largest deformation are selected ($c_{22}^{F}$ and $c_{33}^{F}$) to calculate the overall correction factor $r_{rod}$:

(21) $$r_{rod}=\frac{c_{22}^{F}+c_{33}^{F}}{c_{22}^{\prime \;F}+c_{33}^{\prime \;F}}$$

Using normal instruments, it is difficult to measure the deformation of the measuring rod caused by the gravity. Therefore, we first obtain the compliance coefficients $c_{mn}^{g}$ (m=1,2,3; n=1,2,3) in the simulated environment using the FEA method, and then calculate the approximated actual compliance coefficients $c_{mn}^{g}$ by multiplying $c_{mn}^{\prime \;g}$ by the overall correction factor $r_{rod}$.

(22) $$c_{mn}^{g}\approx r_{rod}c_{mn}^{\prime \;g}=\frac{c_{22}^{F}+c_{33}^{F}}{c_{22}^{\prime \;F}+c_{33}^{\prime \;F}}c_{mn}^{\prime \;g}$$

Finally, all the compliance coefficients used for the deformation compensations are obtained. The unit used for $c_{mn}^{g}$ is the TCP offset value (unit: μm) corresponding to gravitational acceleration *g*. The compliance index $c_{rod}$ is defined and used to evaluate the overall compliance of a measuring rod.

(23) $$c_{rod}=\sum_{m=1}^{3}\sum_{n=1}^{3}c_{mn}^{g}+\sum_{m=1}^{3}\sum_{n=1}^{3}c_{mn}^{F}F$$

where *F* is the magnitude of the measured force.

## 5. Experiments and Results

In this section, calibration experiments were performed on a Staubli TX90 robot using MultiCal, and the results were compared to those obtained using two other devices: a 6D binocular vision measuring system (NDI Polaris Vega, with an accuracy of 3σ=0.2mm) and a measuring arm with a laser scanner (Hexagon AS1, with an overall accuracy of 43 μm).

### 5.1. Measurement of Rod Stiffness

Rod stiffness was determined by both FEA and actual measurement using the device depicted in Figure 7. Gravitational loads of 0.5, 1, 1.5, and 2 kg were applied and the resulting TCP offset measurement was repeated five times for each load. The average TCP offset is plotted in Figure 8. We compare the *Y*-directional compliance coefficients $c_{22}^{F}$ of the 2# (carbon fibre) and 5# (stainless-steel) measuring rods as an example. Furthermore, the theoretical TCP offset (denoted as “5# Calc”) of the 5# measuring rod was calculated based on Equation (15), because the structures of the stainless-steel measuring rods are similar to that of the theoretical model.

As depicted in Figure 8, the carbon fibre measuring rod’s simulated and measured stiffness values are 8.1 and 5.9 times the stainless-steel measuring rod, respectively. The results prove the advantages of using carbon fibre measuring rods in reducing deformation and improving measuring accuracy. The theoretical calculation and FEA results are ideal elastic coefficient equations (y=cx, *c* is the compliance coefficient), and their fitting lines pass through the origin since they are all calculated in an ideal virtual environment. However, both fitting lines of the real measurement results of the 2# and 5# measuring rods intersect the *x*-axis when the load is 0.09 kg to 0.11 kg (about 1 N). This is probably because of the friction in the rails and sliders in the device depicted in Figure 7, making all the actual loads subtract this frictional force, reducing the overall deformation.

It should be noted that the linear condition of the carbon fibre measuring rod is only met under small loads (less than 15 N). As the load increases, non-linear deformations, such as creep, become more pronounced. Therefore, it is crucial to avoid subjecting the rod to excessive loads. This consideration also applies when using carbon fibre rods in other closed-loop devices discussed in Section 1.

Additionally, these observations lead to two implications: Firstly, using a carbon fibre long mounting bracket in TriCal [14] may not be as advantageous as MultiCal since the measuring device is considerably heavier than a precision ball. Secondly, even with the compensation method described in this paper, it may not be effective in applications involving ultra-large robots.

### 5.2. Experiments of Calibration

MultiCal is actually a flexible design scheme that allows users to choose appropriate sensors and design parameters based on the specific needs of the robot being calibrated. In this study, a ceramic ball with a diameter of ϕ30mm and a roundness error of 2 μm was used for the measuring rod. The displacement sensors employed were the ONOSOKKI GS-4830, with a measuring range of 30 mm, a resolution of 1 μm, and an accuracy of 3 μm. Since the first axis of the robot is vertically installed on the workbench, the gravitational acceleration vector $g^{0}$ is equal to $g={\left[0,0,9.81\right]}^{T}$ (unit: m·s${}^{-2}$). The total contact force vector $F^{D}$ of the three displacement sensors was measured as ${\left[1.10,1.25,1.05\right]}^{T}$ (unit: N) using a high-precision force sensor.

The experiment employed a single multi-position fixture with external dimensions of 500 × 300 × 151 mm and a weight of 7.1 kg. This was found to be more accurate than using multiple single-position fixtures attached to the robot workbench, possibly due to insufficient rigidity of the workbench causing slight deformation during measurement and introducing errors. The overall cost of MultiCal was less than 5 K USD, and its measurement accuracy evaluation has been described in previous work [6].

Thirty measurement configurations were selected for each measuring device based on the consideration of calibration accuracy and time efficiency [6]. The configurations were optimized using the observation index (OI) [24] study in RoboDK and MATLAB, and the maximum OI value for each rod was recorded as its theoretical calibration performance. Compliance index $c_{rod}$ and correction factor $r_{rod}$ for each measuring rod were determined through the stiffness measurement described above, and the values are shown in Table 2. The same 30 configurations were used for the carbon fibre and stainless-steel measuring rods with the same structural dimensions ($L_{1}$ and $L_{2}$). Additionally, the correction factor $r_{rod}$ showed that the 3# measuring rod had the greatest difference between the real and simulated stiffness, potentially due to creep deformation of the bonding parts of the rod.

During the calibration process with MultiCal, different measuring rods were installed at the end of the robot to conduct the measurements. The “rough calibration” was performed based on Equation (9), and then calibration with the compensation algorithm and different sets of compliance coefficients was carried out based on Equations (19) and (11). The kinematic parameters and joint stiffness parameters were then obtained by each measuring rod and method.

**Figure 9:**
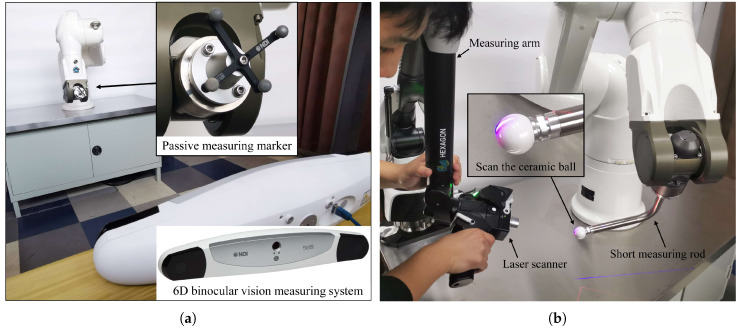
Implement the traditional calibration procedure using (**a**) a 6D binocular vision measuring system and (**b**) a measuring arm with a laser scanner.

Afterward, the MultiCal system was removed, and a 6D binocular vision measuring system (Figure 9) and a measuring arm with a laser scanner were used to conduct traditional calibration procedures, applying the method described in [6]. A short measuring rod was installed at the robot’s end during the laser scanner trial to reduce deformation error, and the surface of the rod’s precision ball was scanned. The spherical centre’s position was obtained through spherical fitting in PolyWorks. The laser scanner and scanning method were also used for validation, with one hundred sets of robot configurations selected in a large portion of the robot’s workspace.

During the validation, the calibrated kinematic and non-kinematic parameters were imported into Equation (9), and only the tool and base parameters were optimized. The final absolute positioning error was then calculated. The nominal kinematic parameters and nominal joint compliance coefficients (which are all zero) were imported into the process, and the average and maximum absolute positioning errors before calibration were calculated as 2.384 mm and 6.571 mm, respectively.

### 5.3. Result and Discussion

After conducting the calibration experiment, the results were obtained (Table 3). The measuring arm with a laser scanner achieved the highest calibration accuracy, while the MultiCal system with various measuring rods and deformation compensation methods also demonstrated good calibration performance, improving the robot’s average and maximum positioning accuracy from 74% to 89% and 69% to 88%, respectively. However, the binocular vision system exhibited the poorest calibration accuracy, likely due to its lower measurement accuracy. Notably, almost all the improvement methods, including the use of carbon fibre measuring rods and deformation compensation methods, improved the MultiCal system’s calibration accuracy compared to the previous work presented in [6]. Specifically, the MultiCal system with the 1# measuring rod and Meas compensation method achieved the highest calibration accuracy, with an average and maximum positioning accuracy of 0.274 mm and 0.838 mm, respectively, similar to that of the laser scanner.

Among the carbon fibre measuring rods, the 1# rod demonstrated the highest calibration accuracy, achieving 3% to 6% and 24% to 27% higher accuracy compared to the 2# and 3# rods, respectively, under the same deformation compensation method. This corresponds to the compliance of the 1# rod only being 83.2% and 22.1% that of the 2# and 3# rods, respectively. Similarly, for the stainless-steel measuring rods, the 5# rod achieved the highest calibration accuracy, 16% to 18% and 26% to 35% higher accuracy than the 4# and 6# rods, respectively, with its compliance being only 80.8% and 31.7% of the 4# and 6# rods, respectively.

Regarding the three compensation methods evaluated, Meas and FEA increased the calibration performance from 6% to 16% compared to the non-compensation method, while Calc showed the poorest performance, yielding a positive effect only when using a long rod. Otherwise, Calc could even reduce the calibration accuracy due to modelling errors.

Surprisingly, the calibration results in this study were more strongly correlated with the stiffness of the measuring rod than the OI value. This could be because the OI value was already relatively high, and the identification error resulting from the singularity of the Jacobi matrix had a smaller effect than the rod deformation error. For example, the “375–600” rods, which had 7% to 13% higher OI values and 2.5 to 4.5 times higher compliance than the other rods, showed 10% to 35% lower calibration accuracy than the other rods.

Overall, using carbon fibre measuring rods with high stiffness (5 to 10 times that of stainless-steel rods of the same size) improved the calibration accuracy from 20% to 39%. In contrast, the deformation compensation methods had a more limited effect, increasing the calibration accuracy from only 6% to 16%. Notably, the compensation methods could only increase the calibration results of the 1# and 2# carbon fibre measuring rods with high stiffness from 6% to 10%. This could be due to the fact that high-rigidity measuring rods reduce the deformation error at the source and that even if the rod deforms, the main part of the TCP offset is downward (due to gravity), so its effect on the calibration accuracy can be largely reduced by optimizing the base parameters $\chi_{B}$ in Equation (9). However, using compensation methods could be essential when calibrating large robots with long or less rigid rods.

**Figure 10:**
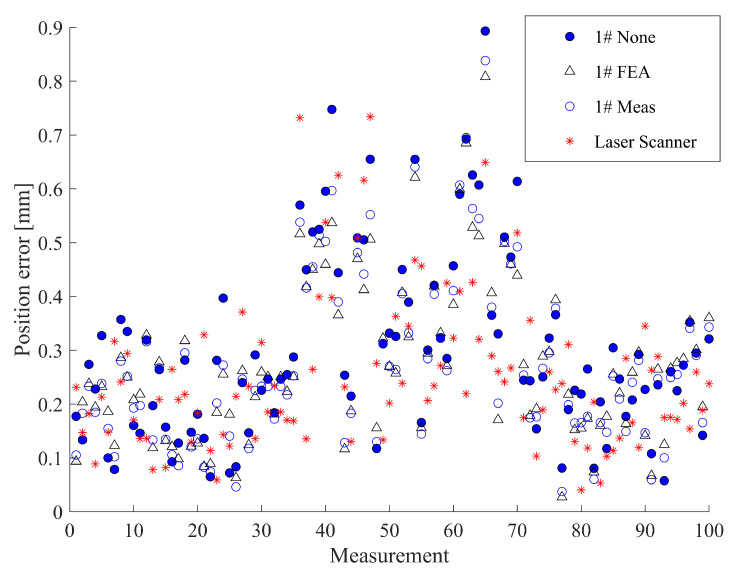
Calibration result of the measuring arm with a laser scanner and the MultiCal with the 1# carbon fibre measuring rod and different deformation compensation methods.

**Figure 11:**
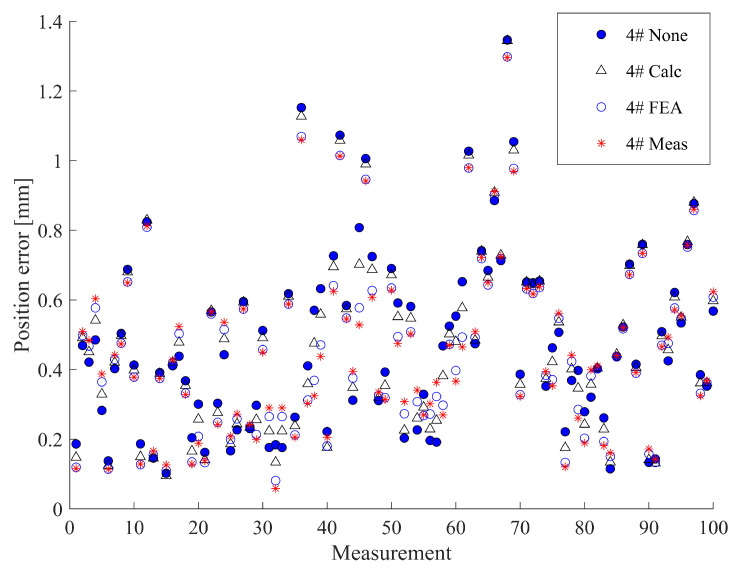
Calibration result of the MultiCal with the 4# stainless-steel measuring rod and different deformation compensation methods.

To facilitate a better comparison of the different compensation methods, we analysed the positioning error distribution obtained by 1# and 4# using different methods (Figure 10 and Figure 11). The results indicate that the error distributions obtained by different methods are very similar since these compensation methods only adjust the measurement coordinates slightly without significantly affecting the overall measurements. Moreover, the primary effect of deformation compensation is to reduce the error of large error points and make the error distribution more uniform. In the case of stainless-steel measuring rods, the results of Meas are comparable to those of FEA since their actual stiffness is close to the simulated stiffness (the rod correction factors $r_{rod}$ are close to one). For carbon fibre measuring rods with a correction factor $r_{rod}$ exceeding 1.2, in most cases, Meas is slightly better than FEA.

In summary, the improved design of MultiCal has significantly enhanced calibration accuracy, approaching the effectiveness of traditional standard measuring tools. However, due to the manual interventions required to switch the clamping position of the measuring device, MultiCal cannot be fully automated such as laser trackers or camera-based systems. Consequently, it does not offer an advantage in large-batch calibration.

Nonetheless, MultiCal remains an excellent choice for on-site calibration and small-batch calibration tasks performed by robot users, after-sales maintenance personnel, and robot researchers and developers. As long as the calibrated robots are not of ultra-large scale and the calibration volume per session is not excessive, MultiCal’s high accuracy and cost-effectiveness make it a valuable solution for these users.

## 6. Conclusions

This paper presents the improved design of an in-contact and on-site calibration device, called MultiCal, which includes a well-designed long carbon fibre measuring rod and a rod deformation compensation algorithm. The redesigned MultiCal offers higher calibration accuracy, low cost (less than 5 K USD), portability, and good robustness. The methods proposed in this paper can be applied to most traditional in-contact calibration devices to overcome their similar drawbacks, particularly when calibrating large robots. The results show that MultiCal achieves a calibration accuracy similar to that of a measuring arm with a laser scanner, with an average and maximum positioning error of 0.274 mm and 0.838 mm, respectively.

This paper also compares the newly-designed carbon fibre measuring rod with the previously-used stainless-steel measuring rod and different deformation compensation methods. The results indicate that the calibration accuracy can be improved from 20% to 39% when using carbon fibre measuring rods and from 6% to 16% when applying the deformation compensation algorithm. The best compensation method involves using the compliance coefficients of the measuring rods obtained from actual measurements and modified FEA results.

Indeed, the effectiveness observed on a single robot model does not necessarily imply its universal applicability to all robot models. Particularly, when dealing with ultra-large robots, which present unique challenges in terms of in-contact measurements, further investigation is required. As part of our future directions, we aim to collect additional data from a diverse range of robot platforms and scenarios to validate and enhance the applicability of our method. Additionally, the optimal location and number of clamping positions for 3D measuring devices require further study.

## Figures and Tables

**Table 1:** MDH parameters of the robot.

Link	θ [${}^{\circ }$]	*d* [mm]	*a* [mm]	α [${}^{\circ }$]	β [${}^{\circ }$]
0–1	$\theta_{1}$	150	$50+\delta a_{1}$	$90+\delta \alpha_{1}$	0
1–2	$\theta_{2}+90+\delta \theta_{2}+c_{2}\tau_{2}$	$-50+\delta d_{2}$	$425+\delta a_{2}$	$0+\delta \alpha_{2}$	$0+\delta \beta_{2}$
2–3	$\theta_{3}+90+\delta \theta_{3}+c_{3}\tau_{3}$	0	$\delta a_{3}$	$90+\delta \alpha_{3}$	0
3–4	$\theta_{4}+\delta \theta_{4}+c_{4}\tau_{4}$	$425+\delta d_{4}$	$\delta a_{4}$	$-90+\delta \alpha_{4}$	0
4–5	$\theta_{5}+\delta \theta_{5}+c_{5}\tau_{5}$	$\delta d_{5}$	$\delta a_{5}$	$90+\delta \alpha_{5}$	0
5–6	$\theta_{6}+\delta \theta_{6}+c_{6}\tau_{6}$	$100+z_{tool}+\delta z_{tool}$	$x_{tool}+\delta x_{tool}$	0	0

**Table 2:** Observability index (OI), compliance index $c_{rod}$, and correction factor $r_{rod}$ of the different measuring rods.

Material	Rod	$L_{1}–L_{2}$	OI	$c_{\mathrm{rod}}$	$r_{\mathrm{rod}}$
carbon fibre	1#	300–450	1.832	57.9	1.226
	2#	150–525	1.721	63.1	1.364
	3#	375–600	1.943	237.3	2.302
stainless steel	4#	300–450	1.832	513.4	1.051
	5#	150–525	1.721	403.7	1.013
	6#	375–600	1.943	1306.2	1.036

**Table 3:** Calibration results of the measuring arm with a laser scanner, 6D binocular vision measuring system, and MultiCal with different measuring rods and deformation compensation methods.

Device and Method		Mean [mm]	Max [mm]	Median [mm]	SD [mm]
1#	None	0.302	0.893	0.265	0.171
carbon	FEA	0.276	0.808	0.256	0.148
fibre	Meas	**0.274**	**0.838**	**0.247**	**0.157**
2#	None	0.312	0.866	0.284	0.162
carbon	FEA	0.290	0.841	0.280	0.159
fibre	Meas	0.286	0.845	0.279	0.153
3#	None	0.414	1.038	0.321	0.204
carbon	FEA	0.364	0.904	0.308	0.186
fibre	Meas	0.362	0.901	0.305	0.186
4#	None	0.475	1.346	0.431	0.254
stainless	Calc	0.467	1.344	0.433	0.251
steel	FEA	0.450	1.298	0.422	0.241
	Meas	0.451	1.296	0.432	0.240
5#	None *	0.392	0.923	0.341	0.207
stainless	Calc	0.404	0.996	0.350	0.215
steel	FEA	0.372	0.907	0.318	0.195
	Meas	0.371	0.901	0.324	0.193
6#	None	0.601	2.012	0.564	0.423
stainless	Calc	0.522	2.120	0.502	0.412
steel	FEA	0.505	1.874	0.482	0.389
	Meas	0.507	1.867	0.480	0.380
Laser scanner		**0.263**	**0.763**	**0.232**	**0.149**
Binocular vision		0.707	2.104	0.590	0.488

* This configuration attained the best calibration result in [6]. The bold number indicates the best performance.

## Data Availability

Data would be available upon request on a personal contact with the corresponding author at the email address: c_zhou@zju.edu.cn.

## Supplementary Materials

The following supporting information can be downloaded at: https://www.mdpi.com/article/10.3390/s23125717/s1, Supplementary Materials: An Improved Design of the MultiCal on-site Calibration Device for Industrial Robots.
