# Supplementary material for: An Improved Design of the MultiCal On-Site Calibration Device for Industrial Robots
Source: Sensors (Basel). 2023 Jun 19;23(12):5717. doi: 10.3390/s23125717 (PMC10305542; doi:10.3390/s23125717)
Supplement: Supplementary file 1 [file sensors-23-05717-s001.zip › suppl.pdf]

# Supplementary Materials: An Improved Design of the MultiCal on-site Calibration Device for Industrial Robots

**Ziwei Wan [1,2], Chunlin Zhou [1,†], Zhaohui Lin [3], Huapeng Yan [4], Weixi Tang [1], Zheng Wang [5], Jun Wu [1]**

## 1. Structural parameters of carbon fiber measuring rods

As depicted in Figure S1, the carbon fiber measuring rod comprises four carbon fiber pipes (pipes A, B, C, and D) and a stainless-steel bending pipe ($\gamma = 90°$). Three different sizes of carbon fiber measuring rods "$L_1$-$L_2$", are defined as "300-450", "150-525", and "375-600". Furthermore, each pipe's length, outer diameter, and thickness are optimized through an FEA strategy. We finally determine that the outer diameter of pipe A, bending pipe, pipe B, pipe C, and pipe D are $\phi$80 mm, $\phi$76 mm, $\phi$72 mm, $\phi$50 mm, and $\phi$30 mm, respectively, and their thickness are 2 mm. The length of each pipe is reported in Table S1:

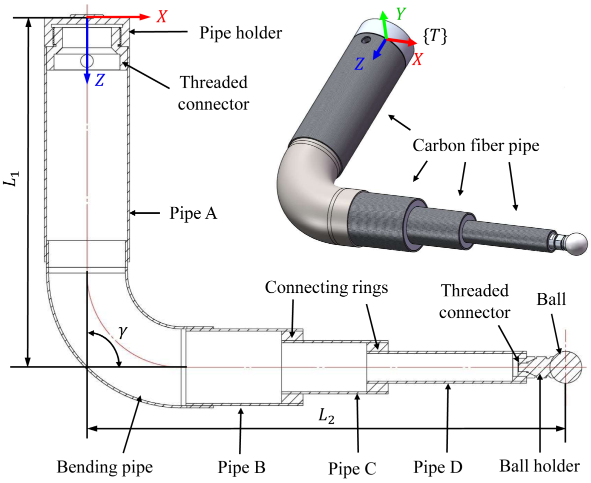


**Figure S1.** Description of the carbon fiber measuring rod

**Table S1.** Length of the carbon fiber measuring rods (Unit: mm)

| | 1#<br>300-450 | 2#<br>150-525 | 3#<br>375-600 |
|---|---|---|---|
| Pipe A ($\phi$80) | 200 | 50 | 275 |
| Pipe B ($\phi$72) | 110 | 145 | 170 |
| Pipe C ($\phi$50) | 100 | 140 | 165 |
| Pipe D ($\phi$30) | 150 | 150 | 175 |

## 2. Fixture calibration

This section describes the fixture calibration process, which aims to obtain the relative positions between different device frames $\{D_j\}$ ($j = 1, 2, \ldots, 5$) and the world frame $\{W\}$ so that the measurement points on each clamping position can be converted into the same frame $\{W\}$. The first step is to establish the device frames $\{D_j\}$ with their $XYZ$ axes parallel to the sensors' measurement axes and their origin being the precision ball's center when the ball makes all the sensors' readings 15 mm (virtual datum point).

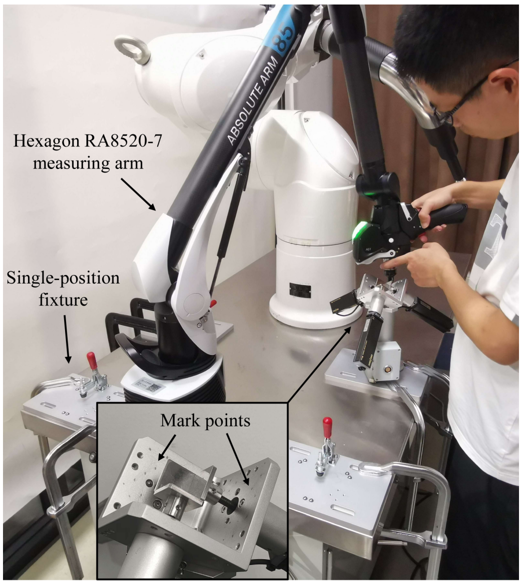


**Figure S2.** Fixture calibration with a Hexagon measuring arm

As shown in Figure S2, similar to the method we used in our previous work [1], we first marked 15 points on each of the triaxial mount's three inner faces, then used a Hexagon RA8520-7 measuring arm to collect the measurement points near these marked points manually and conducted a plane fitting of the obtained point cloud. The inner faces were selected as datum planes for the following reasons. Specifically, first, these surfaces are relatively rigid, and difficult to deform under the measuring arm's probe's measuring force. Second, these inner faces are made by precision machining, and has good shape accuracy (including perpendicularity and flatness). Third, these planes are large enough to ensure the accuracy of plane fitting.

After obtaining the fitting planes of the three inner faces, we obtain the $XY$, $YZ$, and $XZ$ planes of the device frame $\{D_j\}$ using the plane offset command in PolyWorks. The offset distance of each plane was calculated based on Eq. (1):

$$l_{offset} = l_{zero} + l_{target} + \frac{d}{2} \tag{S1}$$

where $l_{zero}$ is the distance from the triaxial mount's inner face to the corresponding square-shaped tip's measuring face when the sensor is at its zero position (measured by the measuring arm), $l_{target}$ is the target reading of each displacement sensor (15 mm), and $d$ is the diameter of the measuring ball (30 mm). Then, the device frame $\{D_j\}$ can be established based on the three offset planes using the frame create command in PolyWorks (Figure S3). After that, we mounted the 3D displacement measuring device on the other clamping positions utilizing the fast-lock mechanisms and obtained the corresponding device frame $\{D_j\}$ with the same method (Figure S3). Finally, the 6D pose parameters between different device frames $\{D_j\}$ and the world frame $\{W\}$ ($\{D_3\}$ is chosen in this paper) were measured in PolyWorks. The measuring arm and the MultiCal were fixed on a rigid workbench using F clamps to ensure high measurement accuracy. Table S2 reports the fixture calibration result of the multi-position fixture finally used in our robot calibration experiment.

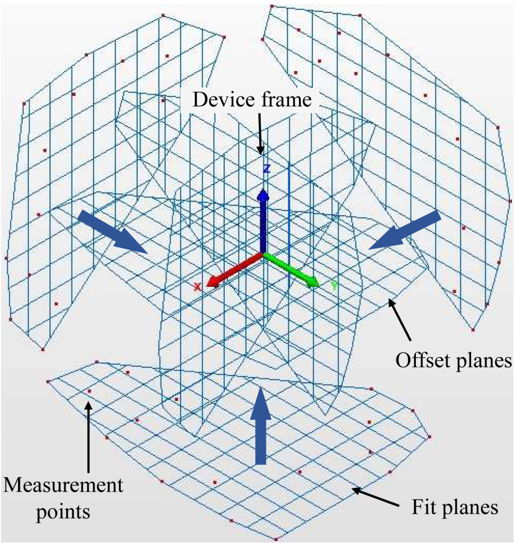


**Figure S3.** Establishing the device frame based on the three offset planes

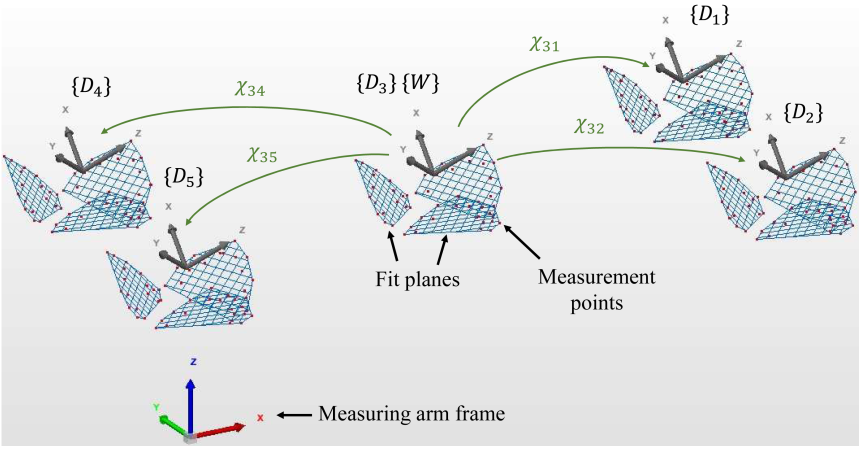


**Figure S4.** Obtaining the 6D pose parameters between different device frames

**Table S2.** 6D pose parameters between different device frames with the world frame

| | $x$ [mm] | $y$ [mm] | $z$ [mm] | $\alpha$ [°] | $\beta$ [°] | $\gamma$ [°] |
|---|---|---|---|---|---|---|
| 1 | 67.201 | −171.035 | 102.240 | 1.028 | 0.156 | −1.523 |
| 2 | −67.107 | −103.118 | 170.004 | −2.215 | 1.211 | 0.594 |
| 4 | 66.806 | 101.566 | −169.920 | 1.253 | −3.860 | 0.125 |
| 5 | −67.269 | 169.149 | −102.141 | 0.251 | −0.134 | 0.951 |

## 3. Theoretical analysis of the rod deformation

This section studies the deformation of the measuring rod under the effect of gravity $\boldsymbol{g}^T$ and measuring force $\boldsymbol{F}^T$ through a theoretical analysis. First, $\boldsymbol{F}^T$ and $\boldsymbol{g}^T$ are decomposed into the $XYZ$ direction of the tool frame $\{T\}$ (Figure S5). According to the linear superposition principle in material mechanics, we analyze the deformation of pipes I and II caused by the $XYZ$ components of $\boldsymbol{F}^T$ and $\boldsymbol{g}^T$, respectively, and then add them up.

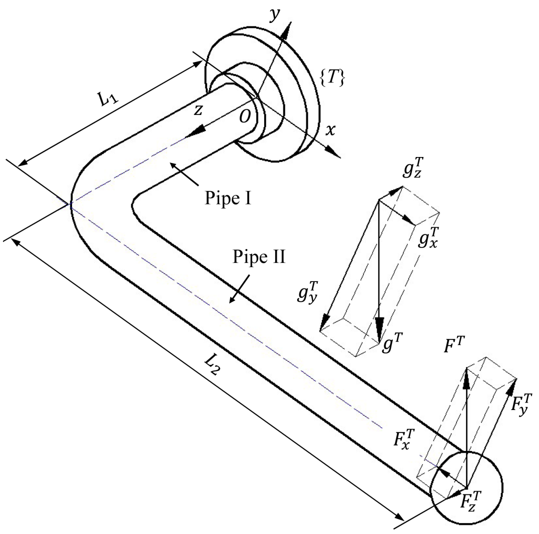


**Figure S5.** Decomposition of the gravity and measuring force in the simplified measuring rod model

### *3.1. Deformation caused by measuring force*

Figure S6 describes the decomposition of the rod deformation (TCP offset $\Delta \boldsymbol{x}^F$) caused by the measuring force. First, the measuring force $\boldsymbol{F}^T$ will cause tension and bending in pipe II, but the TCP offset caused by the tension is nearly negligible. Additionally, $\boldsymbol{F}^T$ also causes bending in pipe I, leading to the positional and angular deviations of pipe II. Besides, the torque imposed by $F_y^T$ will cause pipe I to twist around its axis, resulting in a considerable TCP offset ($\Delta x_{y3}^F$).

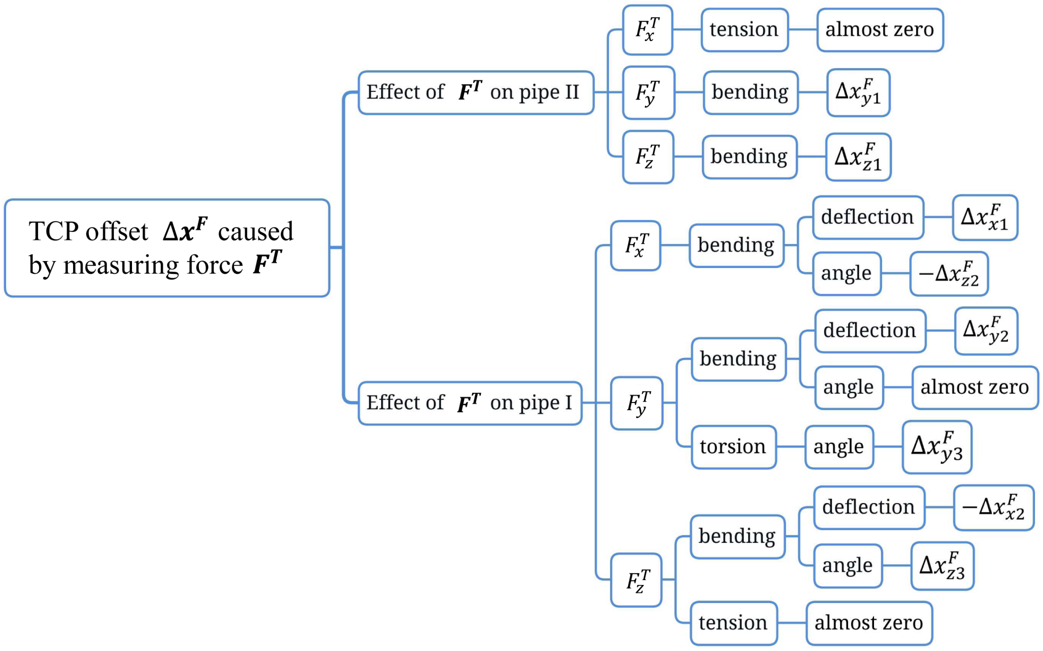


**Figure S6.** Decomposition of the rod deformation (TCP offset $\Delta \boldsymbol{x}^F$) caused by measuring force

The moment of inertia and the polar moment of inertia of the pipe can be calculated by Eq. (2).

$$I_Z = \frac{\pi(D^4 - d^4)}{64} \tag{S2}$$

$$I_P = \frac{\pi(D^4 - d^4)}{32} \tag{S3}$$

where $D$ and $d$ are the outer and inner diameters of the hollow pipe, respectively. Based on the relevant formula in the mechanics of materials, we calculate the TCP offset caused by each part as follows:

$$\Delta x_{y1}^{F} = \frac{F_y^T L_2^3}{3EI_Z} \tag{S4}$$

$$\Delta x_{z1}^{F} = \frac{F_z^T L_2^3}{3EI_Z} \tag{S5}$$

$$\Delta x_{x1}^{F} = \frac{F_x^T L_1^3}{3EI_Z} \tag{S6}$$

$$\Delta x_{z2}^{F} = \frac{F_x^T L_1^2 L_2}{2EI_Z} \tag{S7}$$

$$\Delta x_{y2}^{F} = \frac{F_y^T L_1^3}{3EI_Z} \tag{S8}$$

$$\Delta x_{y3}^{F} = \frac{F_y^T L_1 L_2^2}{GI_P} \tag{S9}$$

$$\Delta x_{x2}^{F} = \frac{F_z^T L_1^2 L_2}{2EI_Z} \tag{S10}$$

$$\Delta x_{z3}^{F} = \frac{F_z^T L_1 L_2^2}{EI_Z} \tag{S11}$$

where $E$ and $G$ are the elastic modulus and shear modulus of the pipes' material, respectively. Then, we obtain the total TCP offset $\Delta \boldsymbol{x}^F$ caused by the measuring force by adding up the above eight TCP offsets in $XYZ$ directions (the signs of these offsets are shown in Figure S6).

$$\Delta x_{x}^{F} = \Delta x_{x1}^{F} - \Delta x_{x2}^{F} = \frac{L_1^3}{3EI_Z} F_x^T - \frac{L_1^2 L_2}{2EI_Z} F_z^T \tag{S12}$$

$$\Delta x_{y}^{F} = \Delta x_{y1}^{F} + \Delta x_{y2}^{F} + \Delta x_{y3}^{F} = \left( \frac{L_1^3 + L_2^3}{3EI_Z} + \frac{L_1 L_2^2}{GI_P} \right) F_y^T \tag{S13}$$

$$\Delta x_{z}^{F} = \Delta x_{z1}^{F} - \Delta x_{z2}^{F} + \Delta x_{z3}^{F} = \left( \frac{L_2^3}{3EI_Z} + \frac{L_1 L_2^2}{EI_Z} \right) F_z^T - \frac{L_1^2 L_2}{2EI_Z} F_x^T \tag{S14}$$

$$\begin{bmatrix} \Delta x_x^F \\ \Delta x_y^F \\ \Delta x_z^F \end{bmatrix} = \begin{bmatrix} \frac{L_1^3}{3EI_Z} & 0 & -\frac{L_1^2 L_2}{2EI_Z} \\ 0 & \frac{L_1^3 + L_2^3}{3EI_Z} + \frac{L_1 L_2^2}{GI_P} & 0 \\ -\frac{L_1^2 L_2}{2EI_Z} & 0 & \frac{L_2^3}{3EI_Z} + \frac{L_1 L_2^2}{EI_Z} \end{bmatrix} \begin{bmatrix} F_x^T \\ F_y^T \\ F_z^T \end{bmatrix} \tag{S15}$$

$$\Delta \boldsymbol{x}^F = \begin{bmatrix} c_{11}^F & 0 & c_{13}^F \\ 0 & c_{22}^F & 0 \\ c_{31}^F & 0 & c_{33}^F \end{bmatrix} \boldsymbol{F}^T = \boldsymbol{C}^F \boldsymbol{F}^T \tag{S16}$$

where $\boldsymbol{C}^F$ is the compliance matrix of the measuring rod for the measuring force and $c_{mn}^F$ ($m = 1, 2, 3$; $n = 1, 2, 3$) are the compliance coefficients of the rod in different directions.

### *3.2. Deformation caused by gravity*

Figure S7 describes the decomposition of the rod deformation (TCP offset $\Delta \boldsymbol{x}^g$) caused by gravity. First, the gravity of pipe I and pipe II will cause tension and bending to themselves, and the TCP offsets caused by the tension are almost zeros. Additionally,

the gravity of pipe II will also bend and twist pipe I (through force and torque) and consequently resulting in the position offset and angular rotation of pipe II.

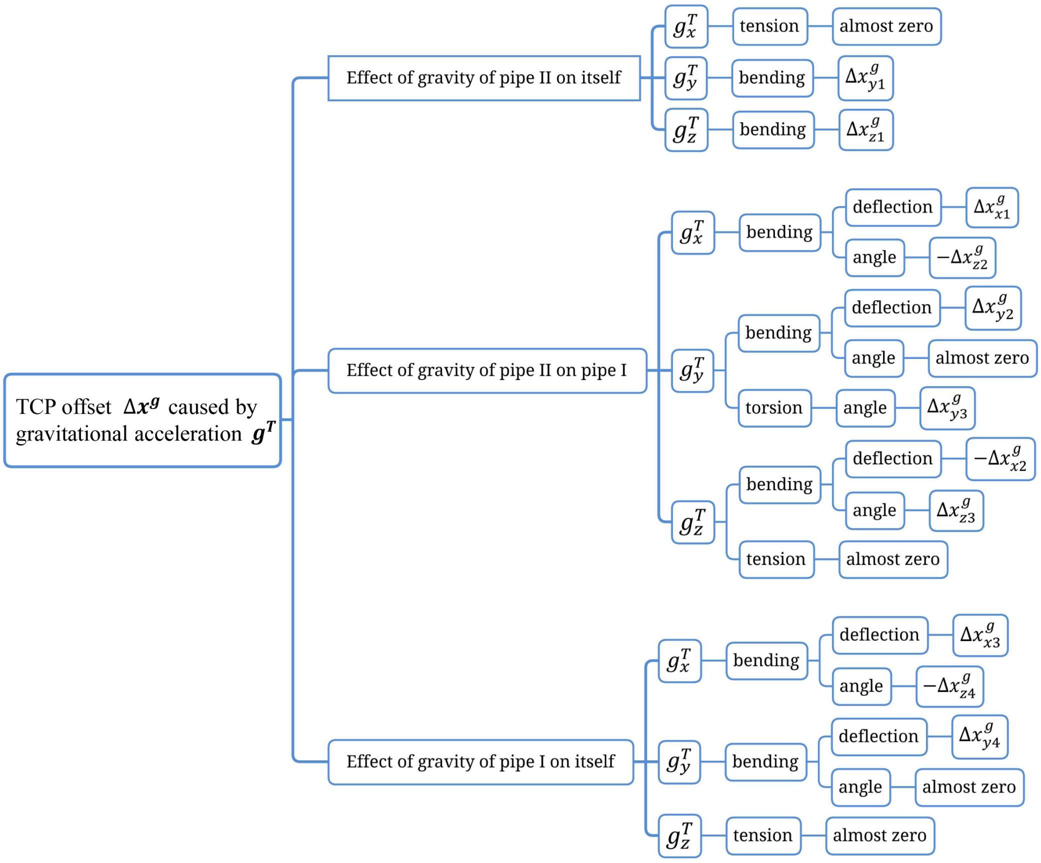


**Figure S7.** Decomposition of the rod deformation (TCP offset $\Delta x^g$) caused by gravity

The uniformly distributed load vector $\boldsymbol{q}^T$ imposed by gravity on the measuring rod in the tool frame $\{T\}$ can be calculated as follows:

$$\boldsymbol{q}^T = \frac{\rho\pi(D^2 - d^2)}{4}\boldsymbol{g}^T \tag{S17}$$

where $\rho$ is the density of the hollow pipe's material, and $\boldsymbol{g}^T$ is the gravitational acceleration vector in the tool frame $\{T\}$. The direction of $\boldsymbol{g}^T$ in each measurement is different since the orientation of the robot's end effector is different. We decompose $\boldsymbol{q}^T$ into the $XYZ$ direction of the tool frame $\{T\}$, namely:

$$\boldsymbol{q}^T = \left[q_x^T, q_y^T, q_z^T\right]^T \tag{S18}$$

Similarly, the TCP offset caused by each part can be calculated as follows:

$$\Delta x_{y1}^g = \frac{q_y^T L_2^4}{8EI_Z} \tag{S19}$$

$$\Delta x_{z1}^g = \frac{q_z^T L_2^4}{8EI_Z} \tag{S20}$$

$$\Delta x_{x1}^g = \frac{q_x^T L_1^3 L_2}{3EI_Z} \tag{S21}$$

$$\Delta x^g_{z2} = \frac{q^T_x L_1^2 L_2^2}{2EI_Z} \tag{S22}$$

$$\Delta x^g_{y2} = \frac{q^T_y L_1^3 L_2}{3EI_Z} \tag{S23}$$

$$\Delta x^g_{y3} = \frac{q^T_y L_1 L_2^3}{2GI_P} \tag{S24}$$

$$\Delta x^g_{x2} = \frac{q^T_z L_1^2 L_2^2}{4EI_Z} \tag{S25}$$

$$\Delta x^g_{z3} = \frac{q^T_z L_1 L_2^3}{2EI_Z} \tag{S26}$$

$$\Delta x^g_{x3} = \frac{q^T_x L_1^4}{8EI_Z} \tag{S27}$$

$$\Delta x^g_{z4} = \frac{q^T_x L_1^3 L_2}{6EI_Z} \tag{S28}$$

$$\Delta x^g_{y4} = \frac{q^T_y L_1^4}{8EI_Z} \tag{S29}$$

Then, we obtain the total TCP offset $\Delta \boldsymbol{x}^g$ caused by the gravitational acceleration by adding up the above eleven TCP offsets in the $XYZ$ directions (the signs of these offsets are shown in Figure S7).

$$\Delta x^g_x = \Delta x^g_{x1} - \Delta x^g_{x2} + \Delta x^g_{x3} = \left(\frac{L_1^3 L_2}{3EI_Z} + \frac{L_1^4}{8EI_Z}\right) q^T_x - \frac{L_1^2 L_2^2}{4EI_Z} q^T_z \tag{S30}$$

$$\Delta x^g_y = \Delta x^g_{y1} + \Delta x^g_{y2} + \Delta x^g_{y3} + \Delta x^g_{y4} = \left(\frac{L_2^4}{8EI_Z} + \frac{L_1^3 L_2}{3EI_Z} + \frac{L_1^4}{8EI_Z} + \frac{L_1 L_2^3}{2GI_P}\right) q^T_y \tag{S31}$$

$$\Delta x^g_z = \Delta x^g_{z1} - \Delta x^g_{z2} + \Delta x^g_{z3} - \Delta x^g_{z4} = \left(\frac{L_2^4}{8EI_Z} + \frac{L_1 L_2^3}{2EI_Z}\right) q^T_z - \left(\frac{L_1^3 L_2}{6EI_Z} + \frac{L_1^2 L_2^2}{2EI_Z}\right) q^T_x \tag{S32}$$

$$\Delta \boldsymbol{x}^g = \begin{bmatrix} \Delta x^g_x \\ \Delta x^g_y \\ \Delta x^g_z \end{bmatrix} = \begin{bmatrix} \frac{L_1^3 L_2}{3EI_Z} + \frac{L_1^4}{8EI_Z} & 0 & -\frac{L_1^2 L_2^2}{4EI_Z} \\ 0 & \frac{L_2^4}{8EI_Z} + \frac{L_1^3 L_2}{3EI_Z} + \frac{L_1^4}{8EI_Z} + \frac{L_1 L_2^3}{2GI_P} & 0 \\ -\frac{L_1^3 L_2}{6EI_Z} - \frac{L_1^2 L_2^2}{2EI_Z} & 0 & \frac{L_2^4}{8EI_Z} + \frac{L_1 L_2^3}{2EI_Z} \end{bmatrix} \begin{bmatrix} q^T_x \\ q^T_y \\ q^T_z \end{bmatrix} \tag{S33}$$

We extract the gravitational acceleration vector $\boldsymbol{g}^T$ in $\boldsymbol{q}^T$, namely:

$$\Delta \boldsymbol{x}^g = \begin{bmatrix} c^g_{11} & 0 & c^g_{13} \\ 0 & c^g_{22} & 0 \\ c^g_{31} & 0 & c^g_{33} \end{bmatrix} \boldsymbol{g}^T = \boldsymbol{C}^g \boldsymbol{g}^T \tag{S34}$$

Then, the final overall TCP offset $\Delta \boldsymbol{x}_{TCP}$ is calculated using Eq. (35).

$$\Delta \boldsymbol{x}_{TCP} = \Delta \boldsymbol{x}^g + \Delta \boldsymbol{x}^F = \boldsymbol{C}^g \boldsymbol{g}^T + \boldsymbol{C}^F \boldsymbol{F}^T \tag{S35}$$

## 4. Measurement of Rod Stiffness

### *4.1. Description of the stiffness measuring device*

We developed a two-dimensional stiffness measuring device (Figure S8) that consists of a loading frame that only moves vertically on rails. A loading plate is positioned at the bottom of the frame to make direct contact with the precision ball of the measuring rod and simulate the measuring force applied to the ball. The force magnitude is determined by weights placed on the top of the loading frame. To reduce frictional resistance, the ball sliders are well lubricated.

Two high-precision displacement sensors (Panasonic TR-1515, measurement range of 15 mm, resolution of 0.5 μm, accuracy of 1.5 μm) are installed orthogonally in the stiffness-measuring device. The sensors have a spherical tip and a square-shaped tip, respectively, for vertical and horizontal measurements of the TCP offset of the measuring rod under the load. The TCP offset measurement method satisfies the Abbe measuring principle [2] and eliminates measuring errors caused by the rotation of the loading plate.

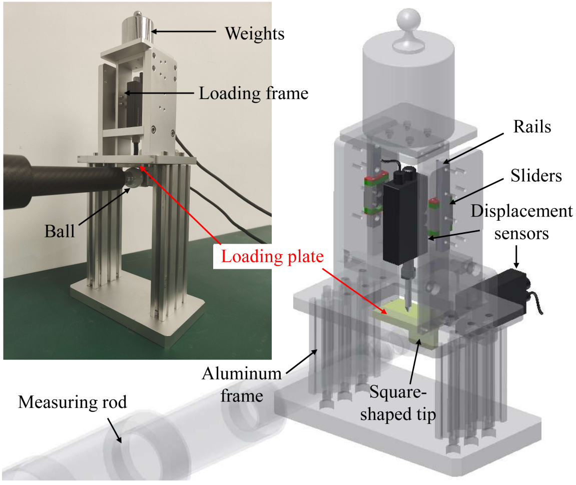


**Figure S8.** Close-up of the two-dimensional stiffness-measuring device

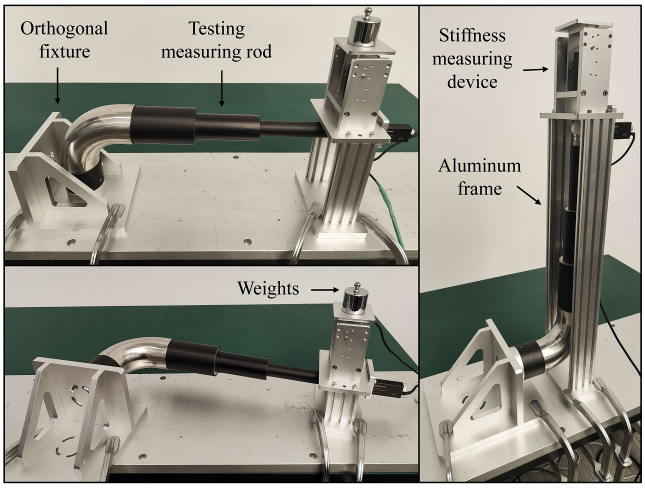


**Figure S9.** Measure the stiffness of the measuring rod in $XYZ$ directions

The device can measure the TCP offset in two directions since the measuring rod will deform significantly in only two directions under the single-direction measuring force (Eq. 16). To measure the rod stiffness in the $XYZ$ directions, we designed an orthogonal fixture (Figure S9) to mount the measuring rod in three different postures. The fixture includes aluminum frames of different heights based on the sizes of the measuring rod.

Before the measurement, the measuring rod is fixed on the orthogonal fixture with its precision ball in contact with both the loading plate and the square-shaped tip, and the displacement sensors are aligned with the tool frame $\{T\}$. The stiffness measuring device and orthogonal fixture are fixed on a large aluminum base plate to ensure the stability.

During measurement, we record the difference between the readings of the two displacement sensors before and after loading, i.e., the TCP offset in both directions. The displacement sensors may not return to their original positions due to system friction and creep deformation of the bonding parts of the measuring rod. To overcome this issue, we manually move the loading frame and square-shaped tip to release them from the ball's surface, then slowly contact them against it. A vibration exciter is used to excite the vibration of all devices and release the dead zone caused by friction. This method has significantly improved the consistency of the measurements.

### 4.2. *Measurement results*

Tables S3, S4, and S5 present the compliance coefficients obtained through theoretical calculation, finite element analysis (FEA), and real measurement, respectively. Note that we only calculate the theoretical compliance coefficients of the stainless-steel measuring rods using Eq. (16) and Eq. (34) since the carbon fiber measuring rod structure is too complex to be modeled theoretically. Besides, the unit used for $c^g_{mn}$ ($m = 1,2,3$; $n = 1,2,3$) is the TCP offset value (unit: µm) corresponding to one gravitational acceleration $g$. All coefficients reserve only one decimal place since MultiCal has only a µm-level accuracy.

**Table S3.** Compliance coefficients obtained by the theoretical calculation

| Compliance terms | gravity [µm] | | | | | measuring force [µm N$^{-1}$] | | | | |
|---|---|---|---|---|---|---|---|---|---|---|
| | $c^g_{11calc}$ | $c^g_{31calc}$ | $c^g_{22calc}$ | $c^g_{13calc}$ | $c^g_{33calc}$ | $c^F_{11calc}$ | $c^F_{31calc}$ | $c^F_{22calc}$ | $c^F_{13calc}$ | $c^F_{33calc}$ |
| 4# | 20.6 | −45.4 | 104.0 | −18.6 | 76.5 | 2.7 | −6.1 | 32.2 | −6.1 | 27.3 |
| 5# | 2.7 | −13.8 | 90.9 | −6.3 | 82.9 | 0.3 | −1.8 | 28.6 | −1.8 | 26.8 |
| 6# | 53.0 | −124.6 | 304.1 | −51.5 | 230.9 | 5.3 | −12.6 | 72.1 | −12.6 | 61.9 |

**Table S4.** Compliance coefficients obtained by the finite element analysis (FEA)

| Compliance terms | gravity [µm] | | | | | measuring force [µm N$^{-1}$] | | | | |
|---|---|---|---|---|---|---|---|---|---|---|
| | $c^g_{11FEA}$ | $c^g_{31FEA}$ | $c^g_{22FEA}$ | $c^g_{13FEA}$ | $c^g_{33FEA}$ | $c^F_{11FEA}$ | $c^F_{31FEA}$ | $c^F_{22FEA}$ | $c^F_{13FEA}$ | $c^F_{33FEA}$ |
| 1# | 1.9 | −4.9 | 9.2 | −2.1 | 10.5 | 0.2 | −0.5 | 3.6 | −0.8 | 4.8 |
| 2# | 0.5 | −2.8 | 9.0 | −1.4 | 12.3 | 0.1 | −0.3 | 3.8 | −0.6 | 5.6 |
| 3# | 3.6 | −9.5 | 20.4 | −4.0 | 23.9 | 0.3 | −0.8 | 6.8 | −1.3 | 9.1 |
| 4# | 19.3 | −44.3 | 130.0 | −25.6 | 106.0 | 2.9 | −6.3 | 35.0 | −7.7 | 31.1 |
| 5# | 2.7 | −14.6 | 113.8 | −12.5 | 118.5 | 0.4 | −2.1 | 31.1 | −3.6 | 32.1 |
| 6# | 49.9 | −121.0 | 371.5 | −65.8 | 300.3 | 5.5 | −12.8 | 76.5 | −15.2 | 69.0 |

**Table S5.** Compliance coefficients obtained by the real measurements

| Compliance terms | $r_{rod}$ | gravity [µm] | | | | | measuring force [µm N$^{-1}$] | | | | |
|---|---|---|---|---|---|---|---|---|---|---|---|
| | | $c^g_{11meas}$ | $c^g_{31meas}$ | $c^g_{22meas}$ | $c^g_{13meas}$ | $c^g_{33meas}$ | $c^F_{11meas}$ | $c^F_{31meas}$ | $c^F_{22meas}$ | $c^F_{13meas}$ | $c^F_{33meas}$ |
| 1# | 1.226 | 2.3 | −5.9 | 11.3 | −2.6 | 12.9 | 0.1 | −0.8 | 4.2 | −0.4 | 6.1 |
| 2# | 1.364 | 0.7 | −3.8 | 12.3 | −1.9 | 16.8 | 0.1 | −0.6 | 5.3 | −0.5 | 7.5 |
| 3# | 2.302 | 8.3 | −21.9 | 47.0 | −9.2 | 55.0 | 6.5 | −2.4 | 15.3 | −3.2 | 21.3 |
| 4# | 1.051 | 20.3 | −46.6 | 136.7 | −26.9 | 111.5 | 2.9 | −6.5 | 36.5 | −8.1 | 33.0 |
| 5# | 1.013 | 2.8 | −14.8 | 115.2 | −12.7 | 120.0 | 0.3 | −2.2 | 31.5 | −3.7 | 32.5 |
| 6# | 1.036 | 51.7 | −125.4 | 385.0 | −68.2 | 311.2 | 5.5 | −12.5 | 78.7 | −16.3 | 72.1 |

## References

1. Wan, Z.; Zhou, C.; Zhang, H.; Wu, J. Development of an onsite calibration device for robot manipulators. *Frontiers of Information Technology & Electronic Engineering* **2023**, *24*, 217–230. https://doi.org/10.1631/fitee.2200172.
2. Fan, K.C.; Liu, C.L.; Wu, P.T.; Chen, Y.C.; Wang, W.L. The Structure Design of a Micro-precision CMM with Abbé Principle. In *Proceedings of the 35$^{th}$ International MATADOR Conference*; Springer London, 2007; pp. 297–300. https://doi.org/10.1007/978-1-84628-988-0_67.
