# Supplementary figures and images for: An Improved Design of the MultiCal On-Site Calibration Device for Industrial Robots
Source: Sensors (Basel). 2023 Jun 19;23(12):5717. doi: 10.3390/s23125717 (PMC10305542; doi:10.3390/s23125717)

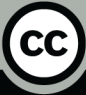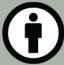

BY

Supplement: Supplementary file 1 [file sensors-23-05717-s001.zip › Definitions/logo-ccby-eps-converted-to.pdf]

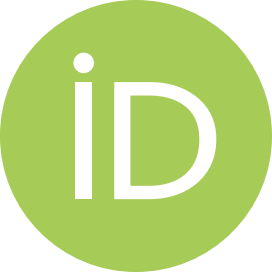

Supplement: Supplementary file 1 [file sensors-23-05717-s001.zip › Definitions/logo-orcid.pdf]

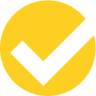

check for  
updates

Supplement: Supplementary file 1 [file sensors-23-05717-s001.zip › Definitions/logo-updates-eps-converted-to.pdf]

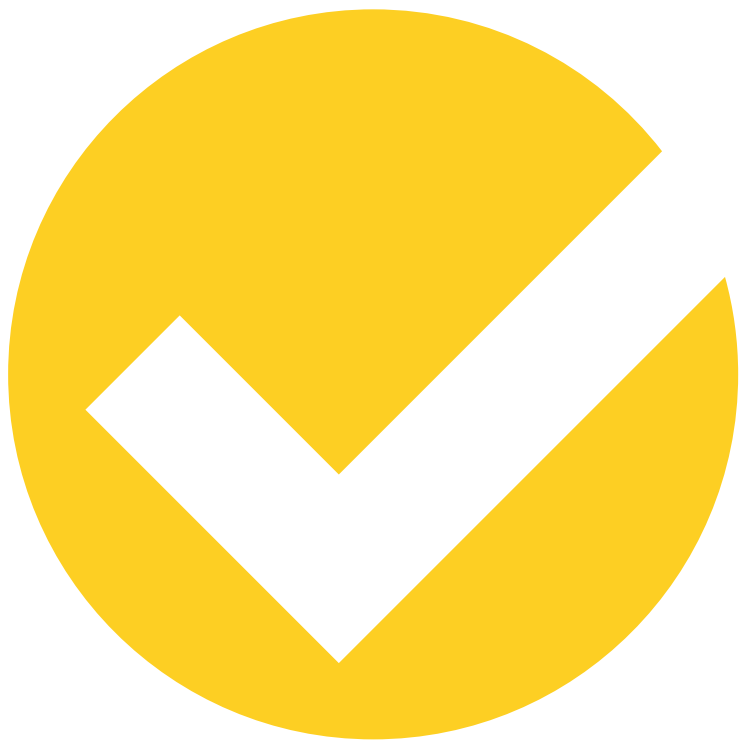

check for  
updates

Supplement: Supplementary file 1 [file sensors-23-05717-s001.zip › Definitions/logo-updates.pdf]

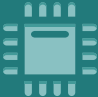

*sensors*

Supplement: Supplementary file 1 [file sensors-23-05717-s001.zip › Definitions/sensors-logo-eps-converted-to.pdf]

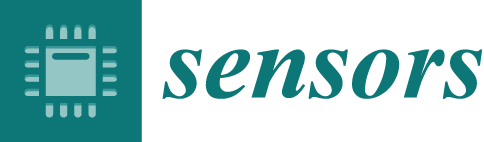

Supplement: Supplementary file 1 [file sensors-23-05717-s001.zip › Definitions/sensors-logo.png]

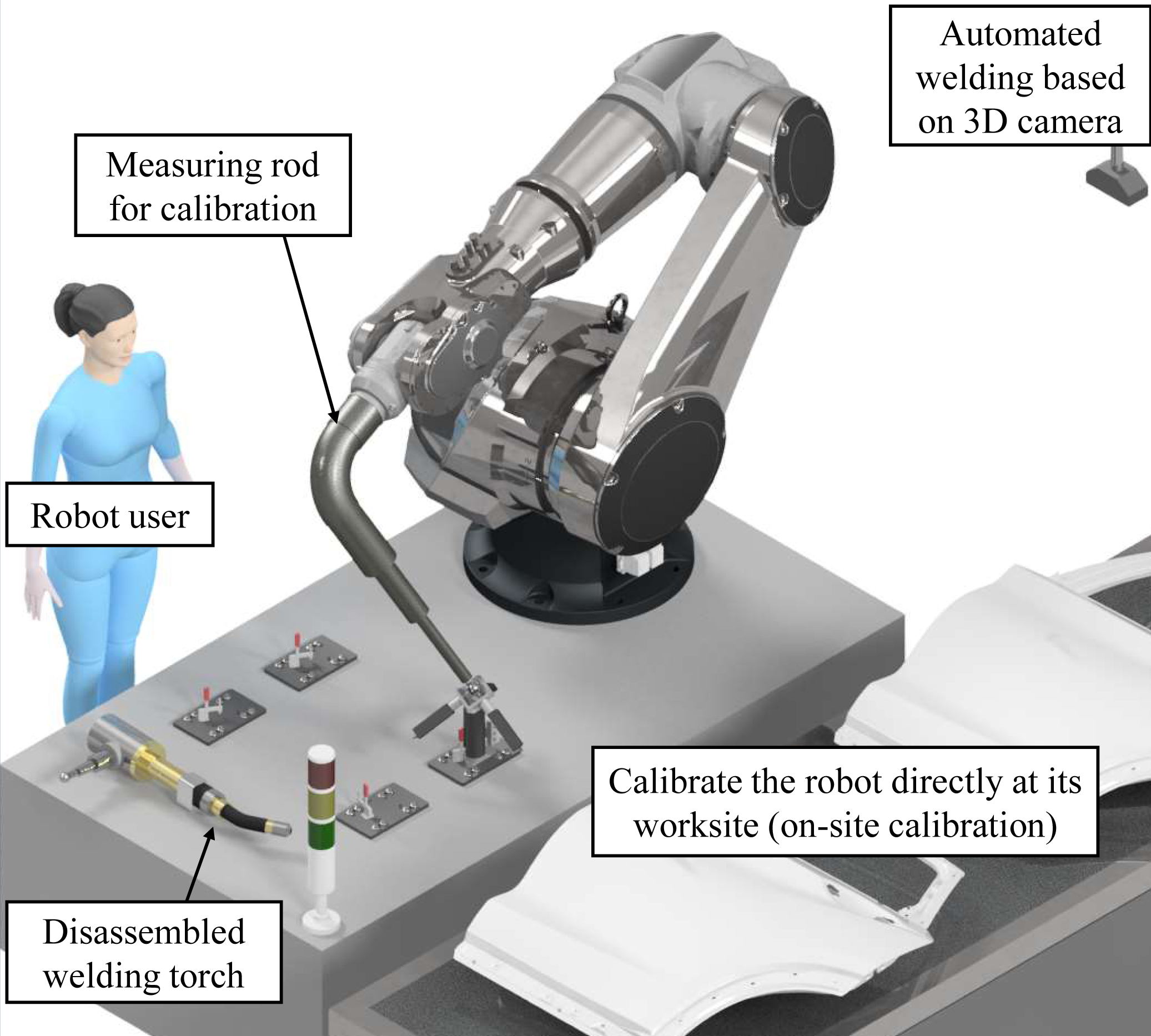

Supplement: Supplementary file 1 [file sensors-23-05717-s001.zip › Figures/Fig1-a.png]

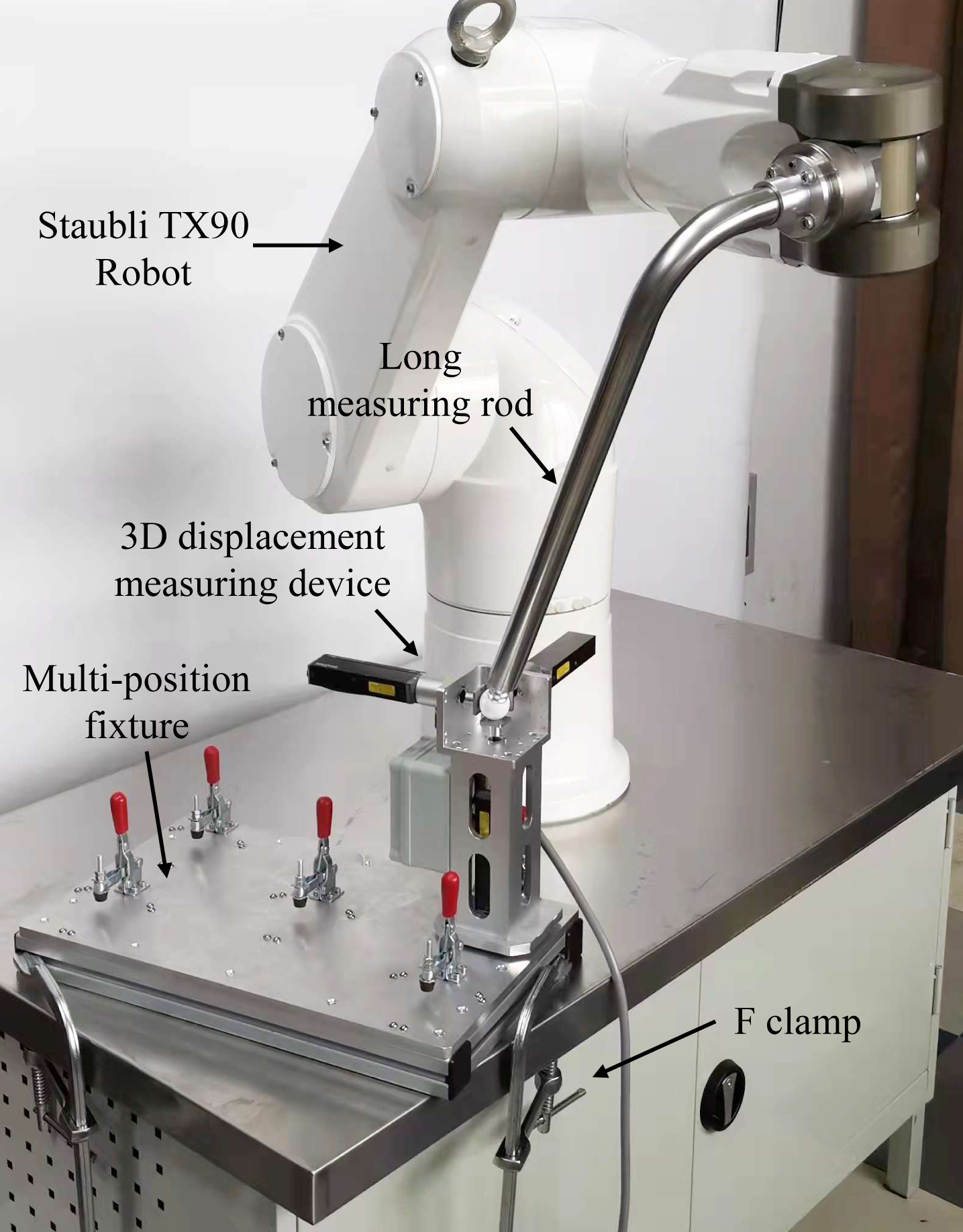

Supplement: Supplementary file 1 [file sensors-23-05717-s001.zip › Figures/Fig1-b.png]

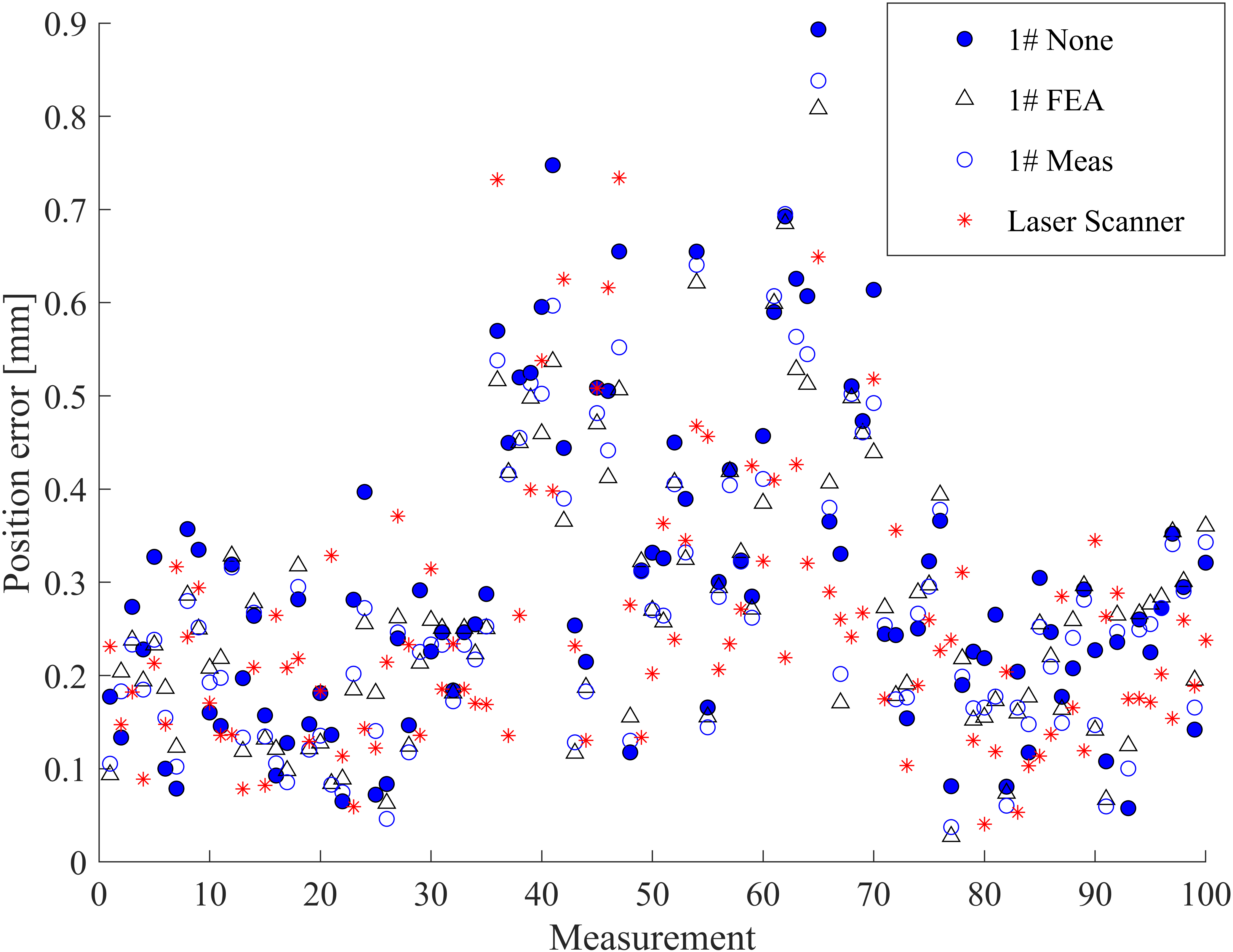

Supplement: Supplementary file 1 [file sensors-23-05717-s001.zip › Figures/Fig10.png]

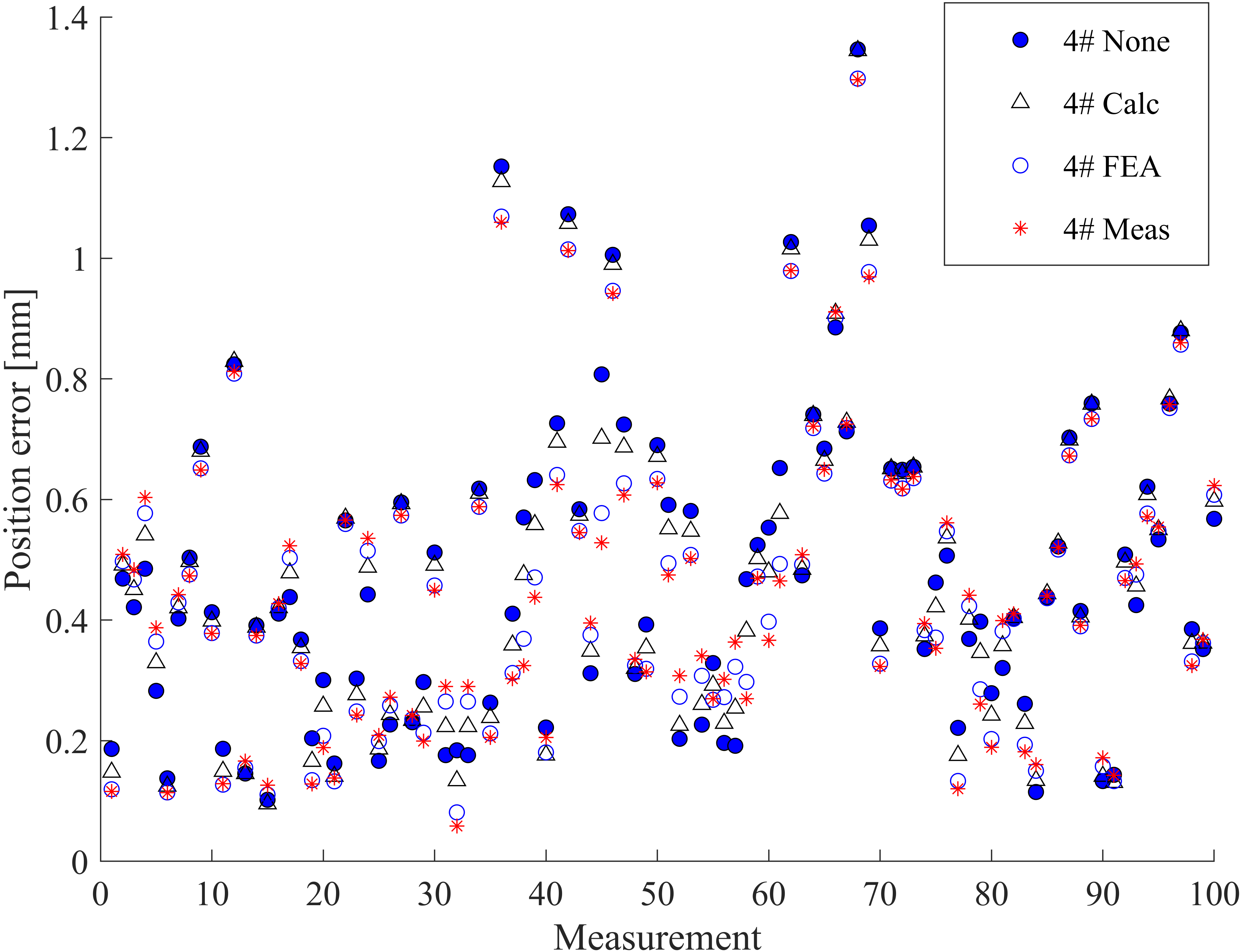

Supplement: Supplementary file 1 [file sensors-23-05717-s001.zip › Figures/Fig11.png]

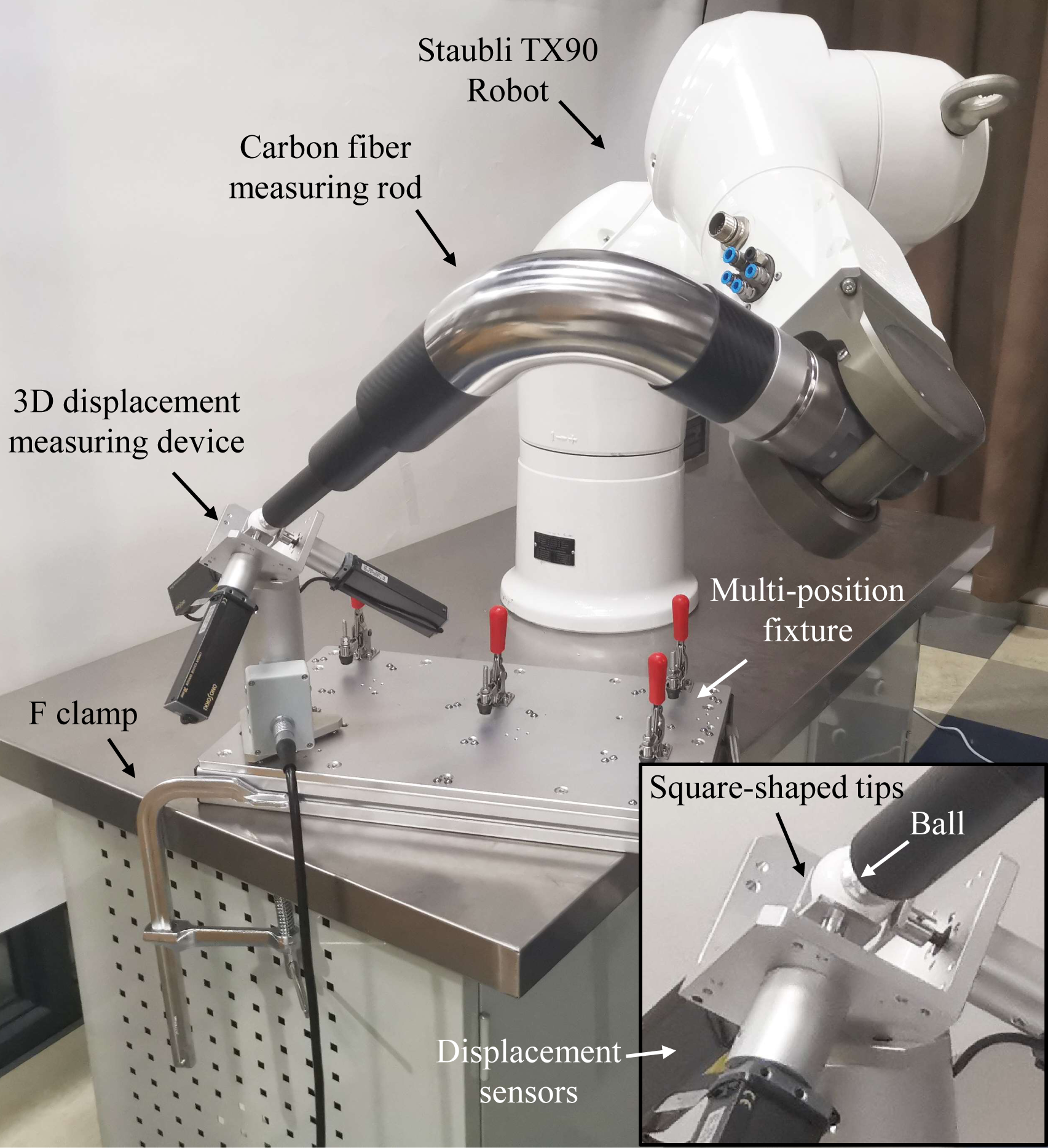

Supplement: Supplementary file 1 [file sensors-23-05717-s001.zip › Figures/Fig2-a.png]

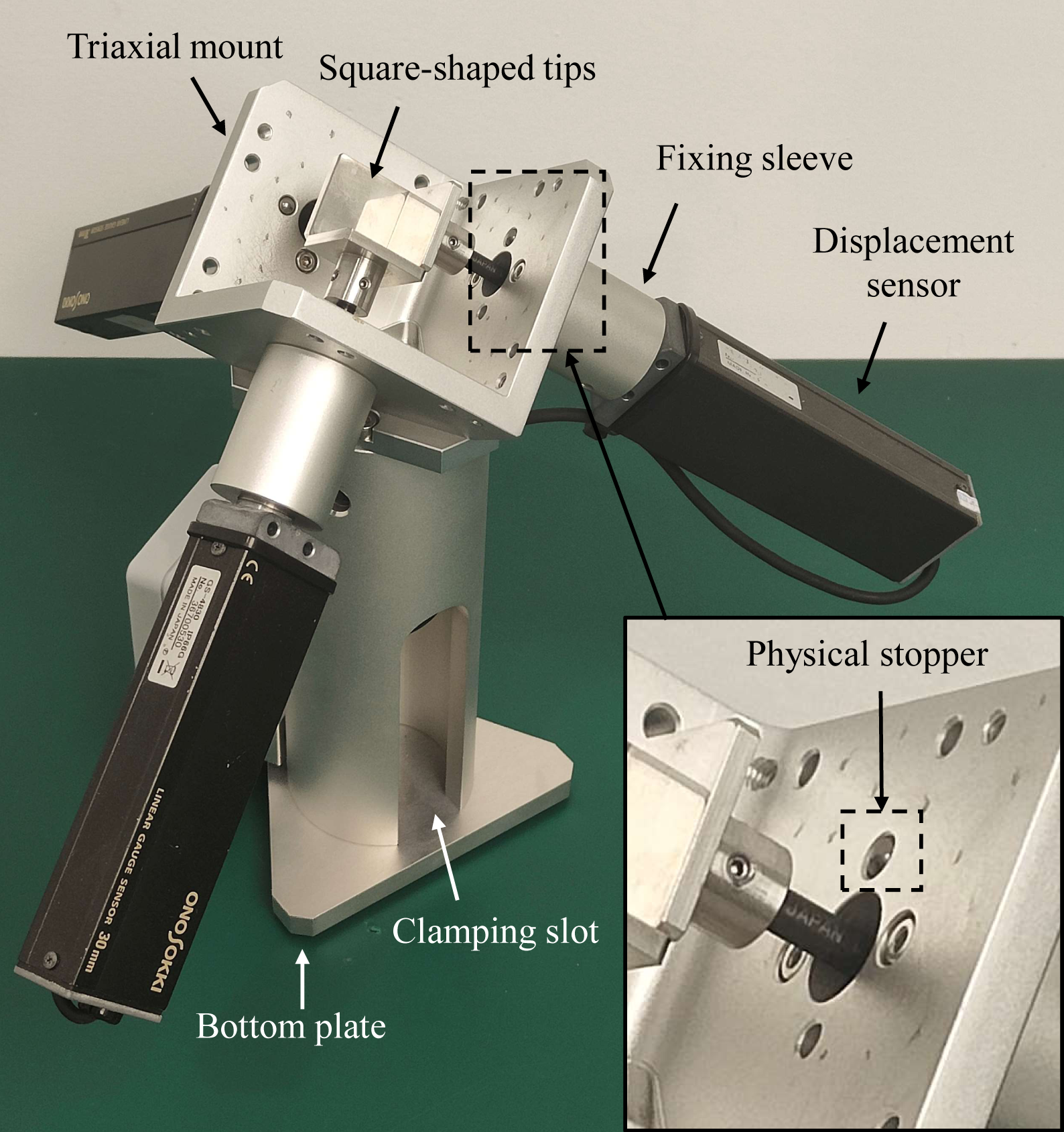

Supplement: Supplementary file 1 [file sensors-23-05717-s001.zip › Figures/Fig2-b.png]

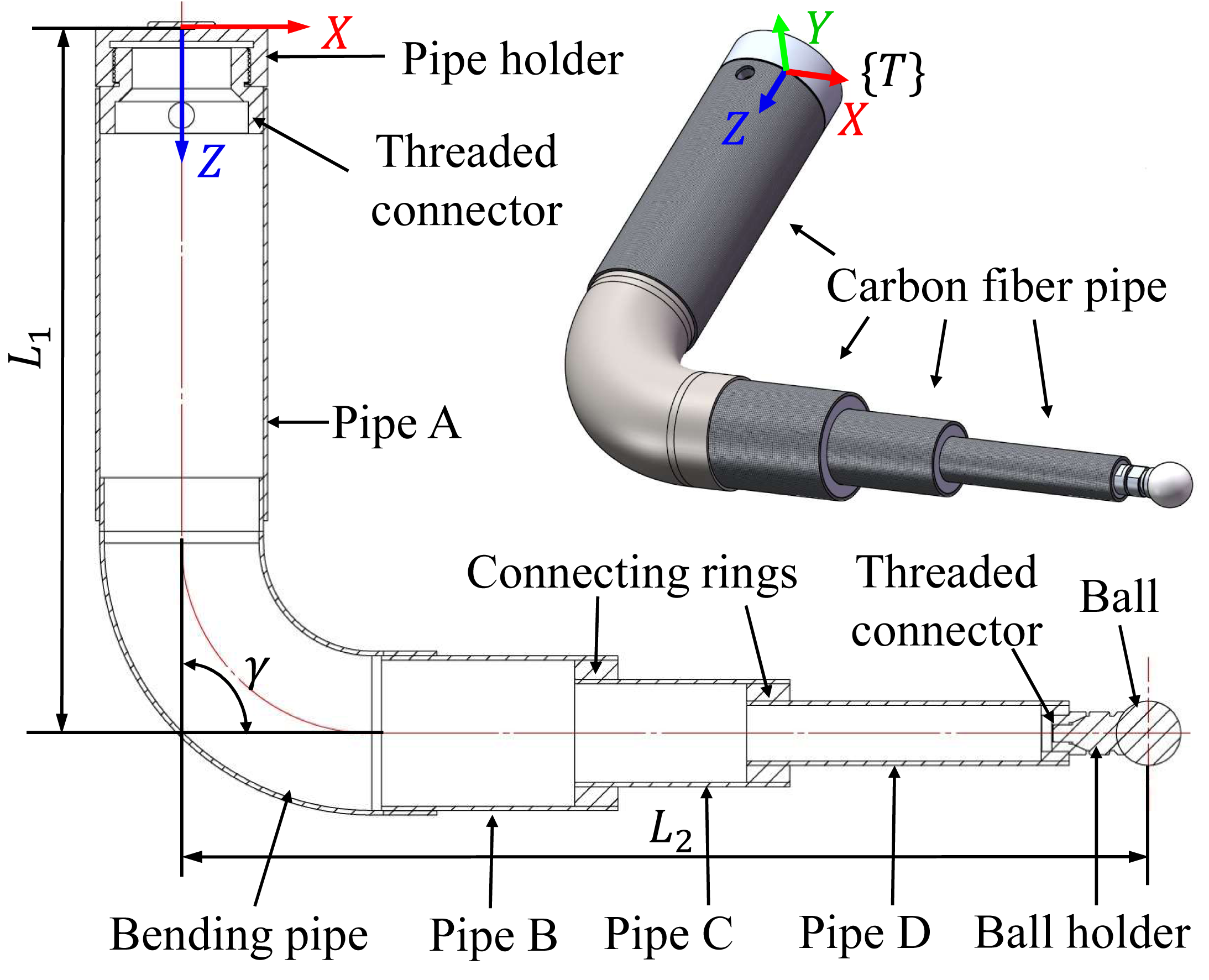

Supplement: Supplementary file 1 [file sensors-23-05717-s001.zip › Figures/Fig3-a.png]

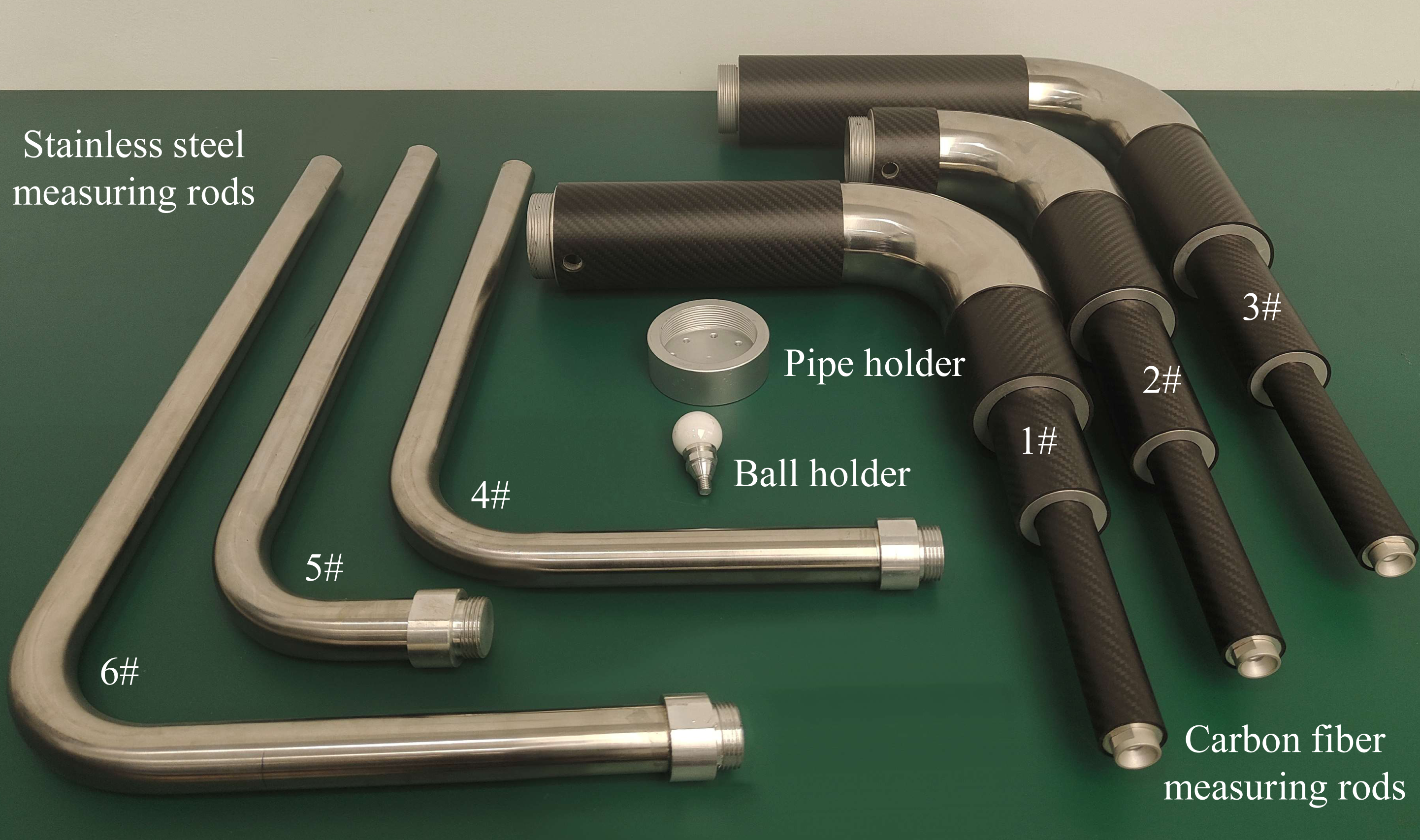

Supplement: Supplementary file 1 [file sensors-23-05717-s001.zip › Figures/Fig3-b.png]

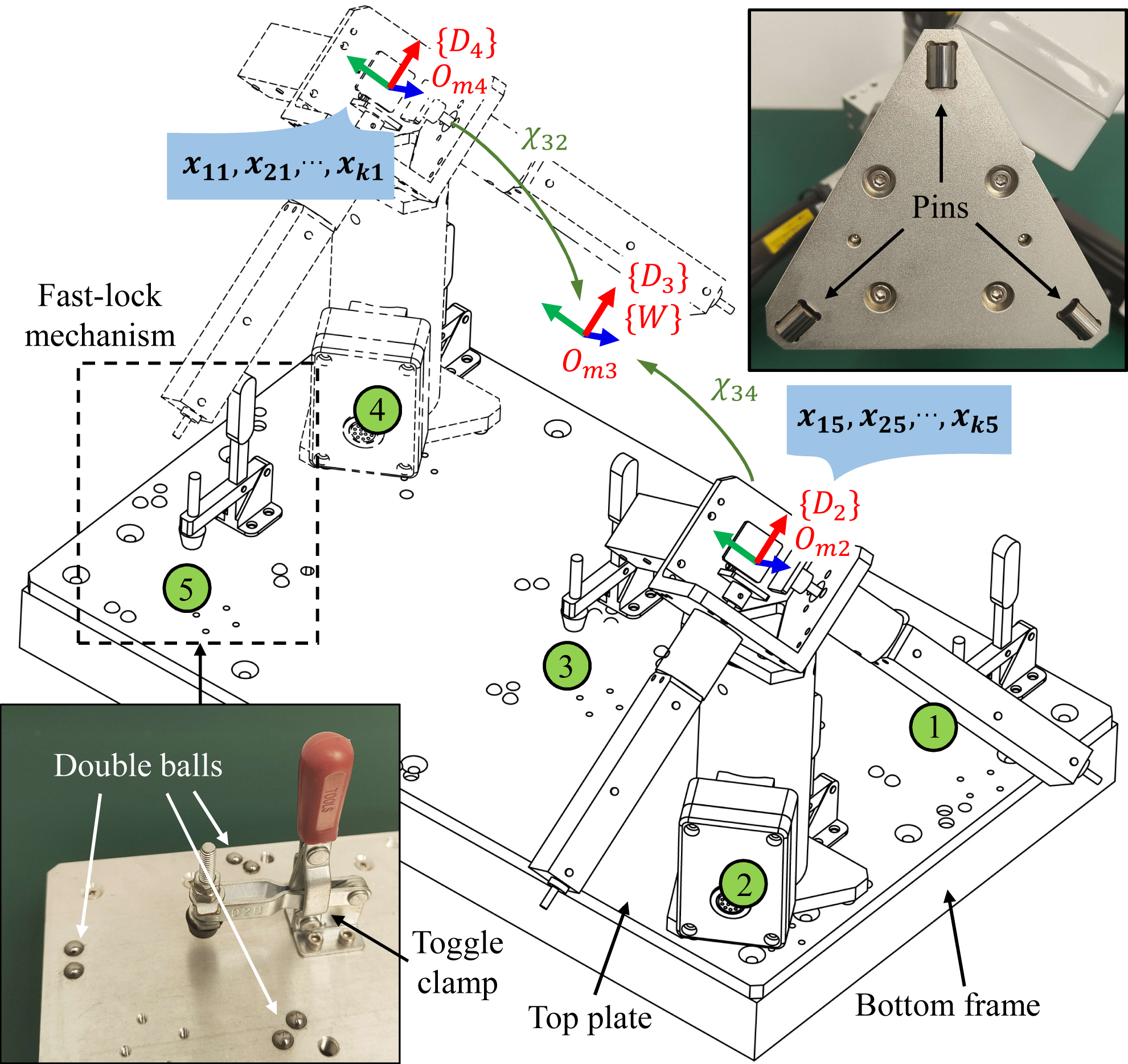

Supplement: Supplementary file 1 [file sensors-23-05717-s001.zip › Figures/Fig4.png]

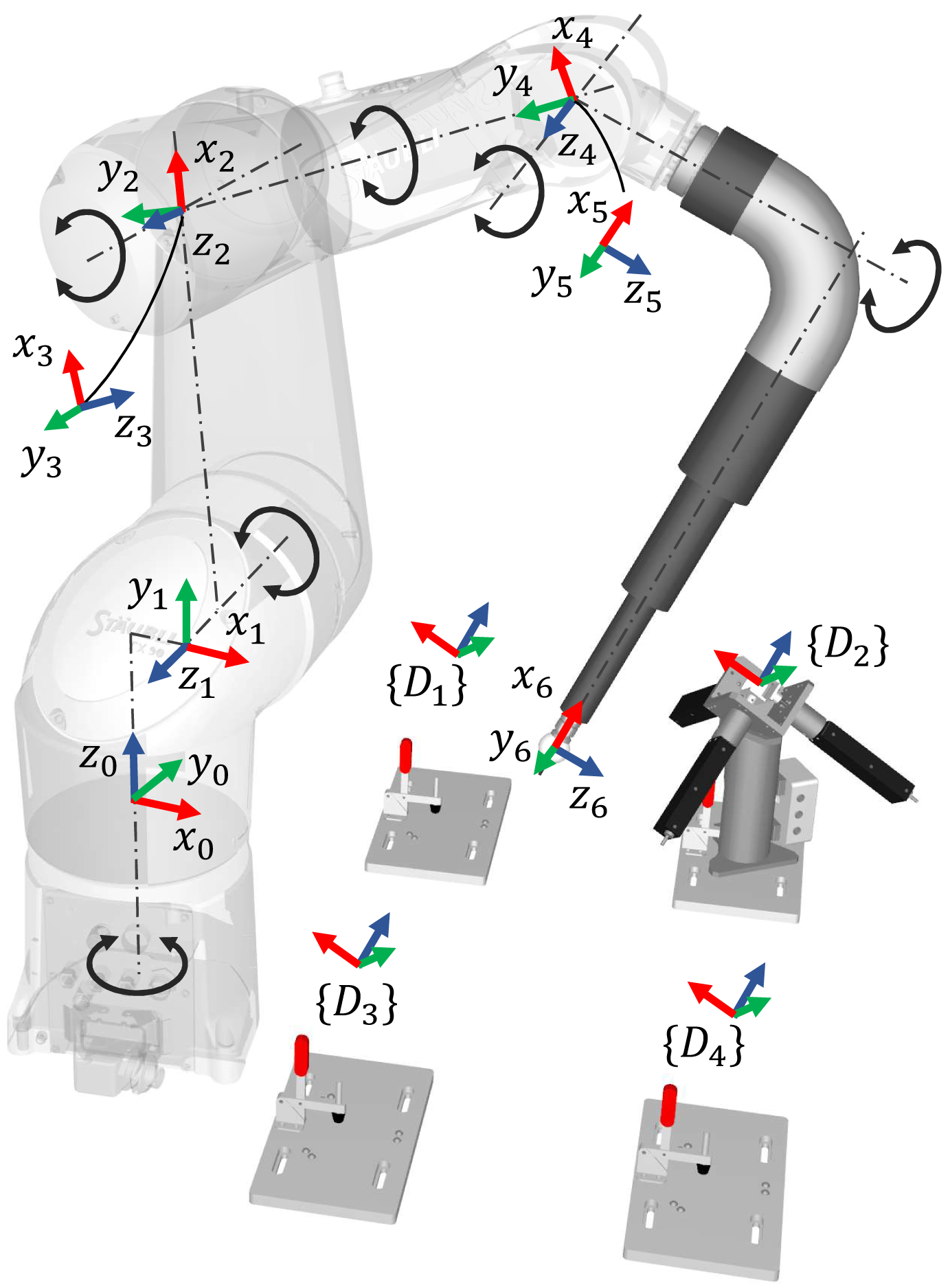

Supplement: Supplementary file 1 [file sensors-23-05717-s001.zip › Figures/Fig5.png]

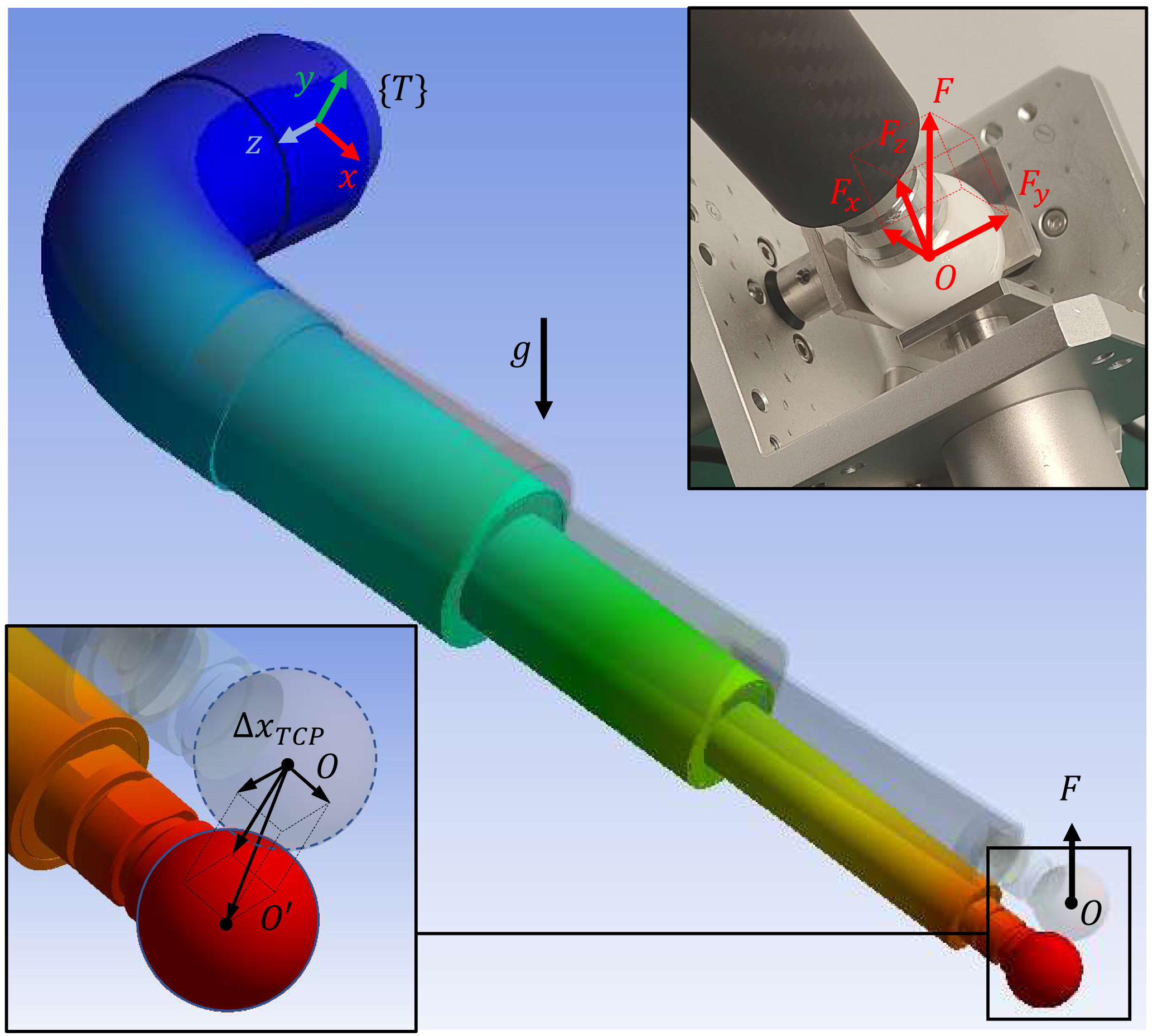

Supplement: Supplementary file 1 [file sensors-23-05717-s001.zip › Figures/Fig6-a.png]

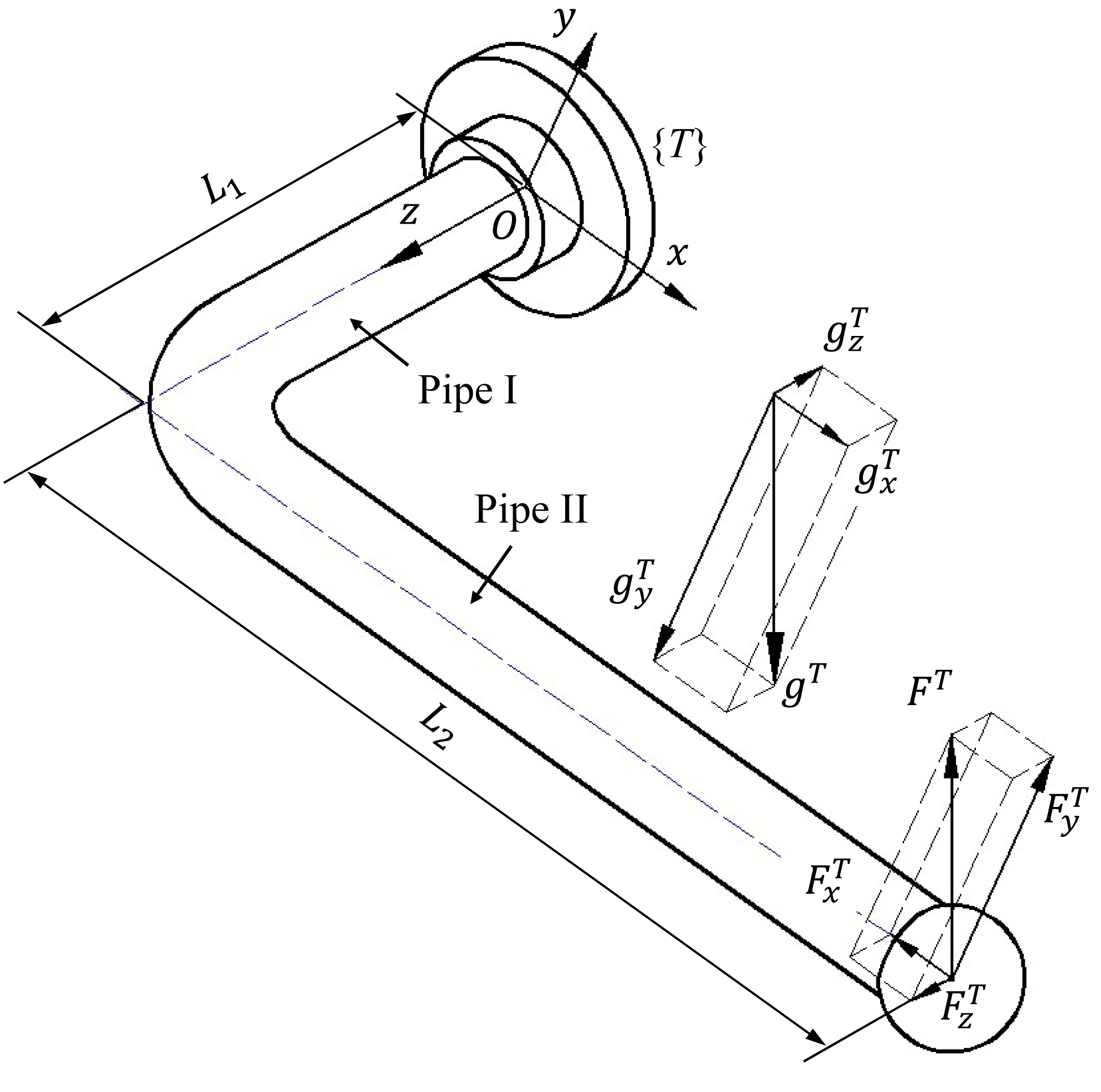

Supplement: Supplementary file 1 [file sensors-23-05717-s001.zip › Figures/Fig6-b.png]

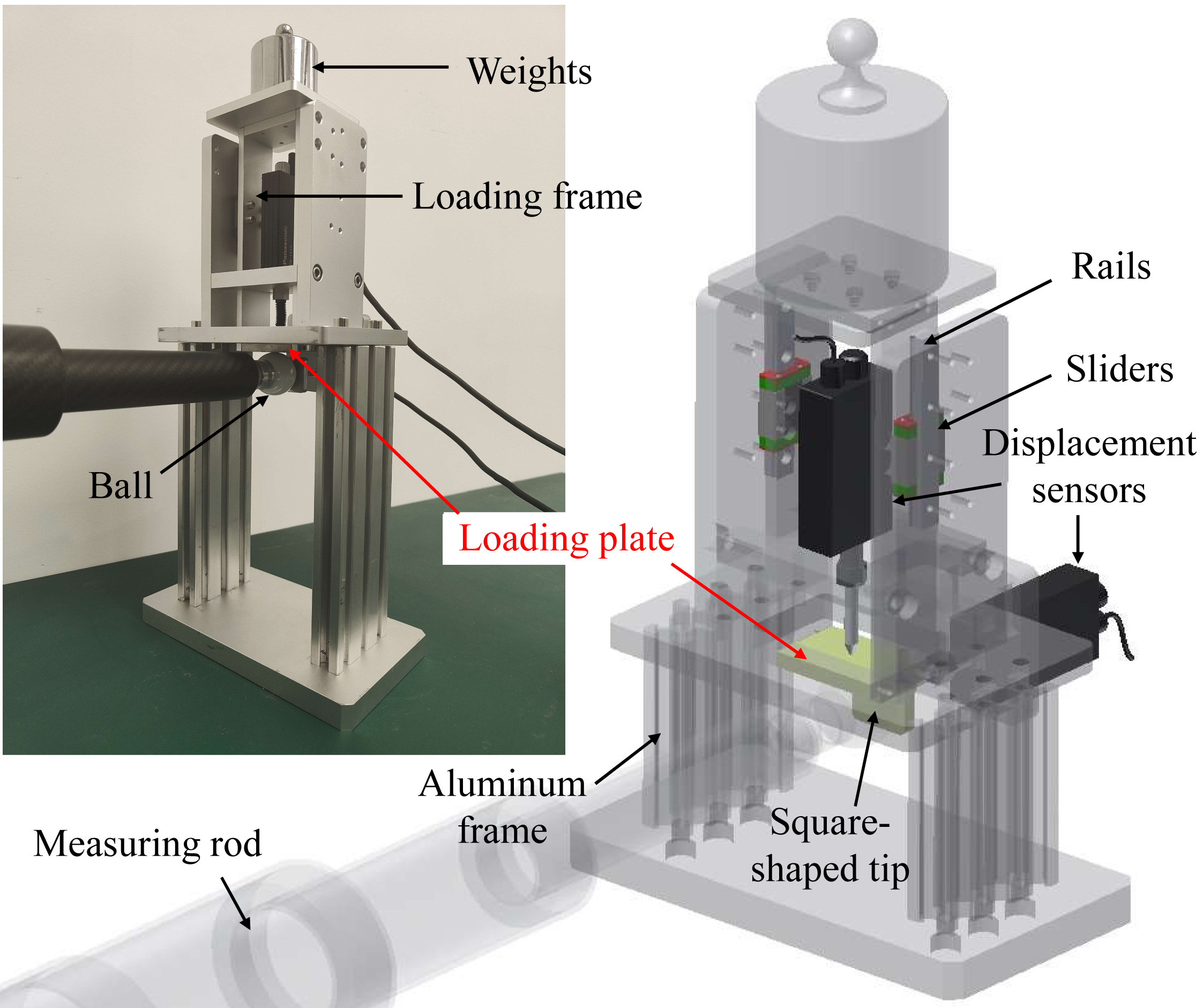

Supplement: Supplementary file 1 [file sensors-23-05717-s001.zip › Figures/Fig7.png]

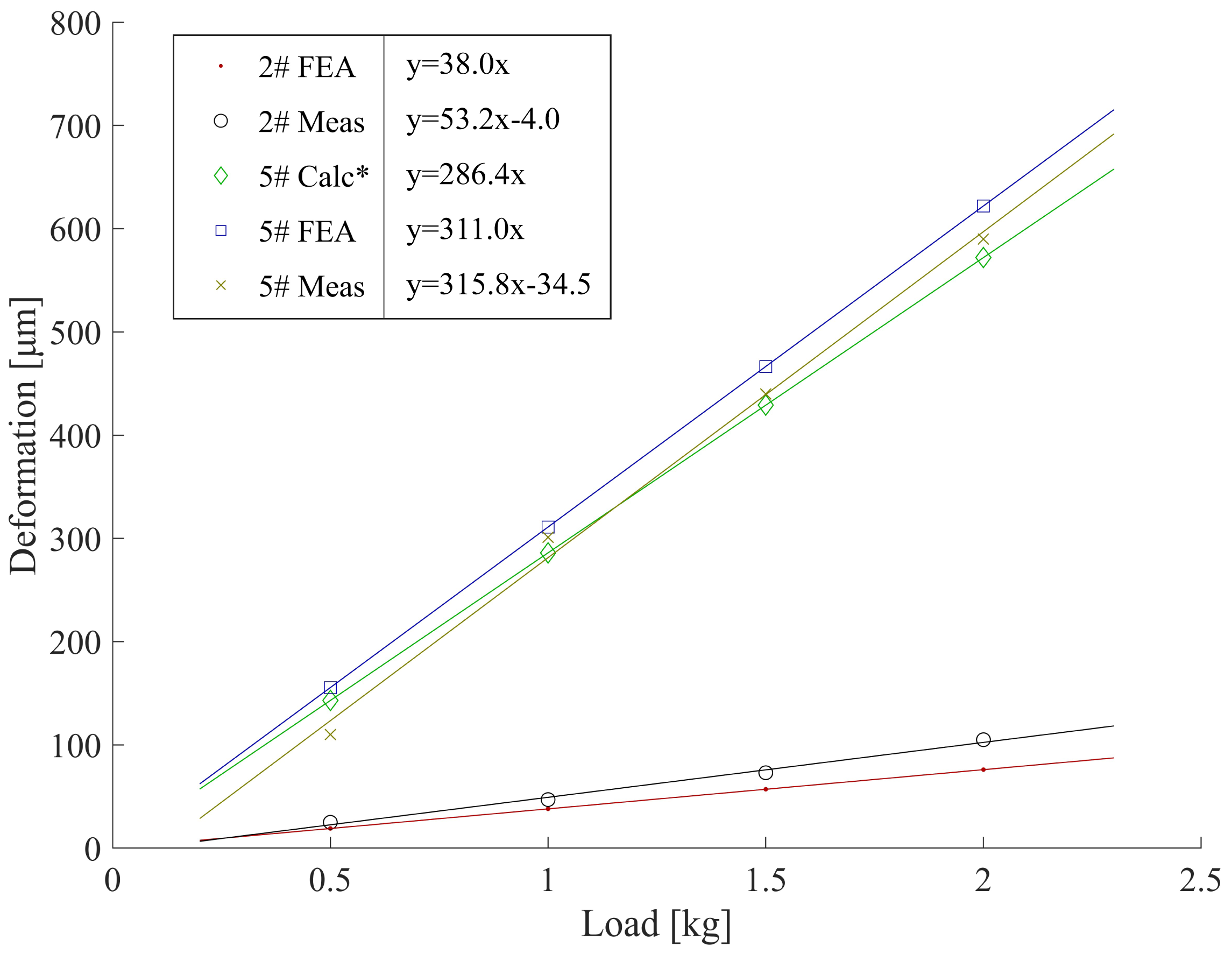

Supplement: Supplementary file 1 [file sensors-23-05717-s001.zip › Figures/Fig8.png]

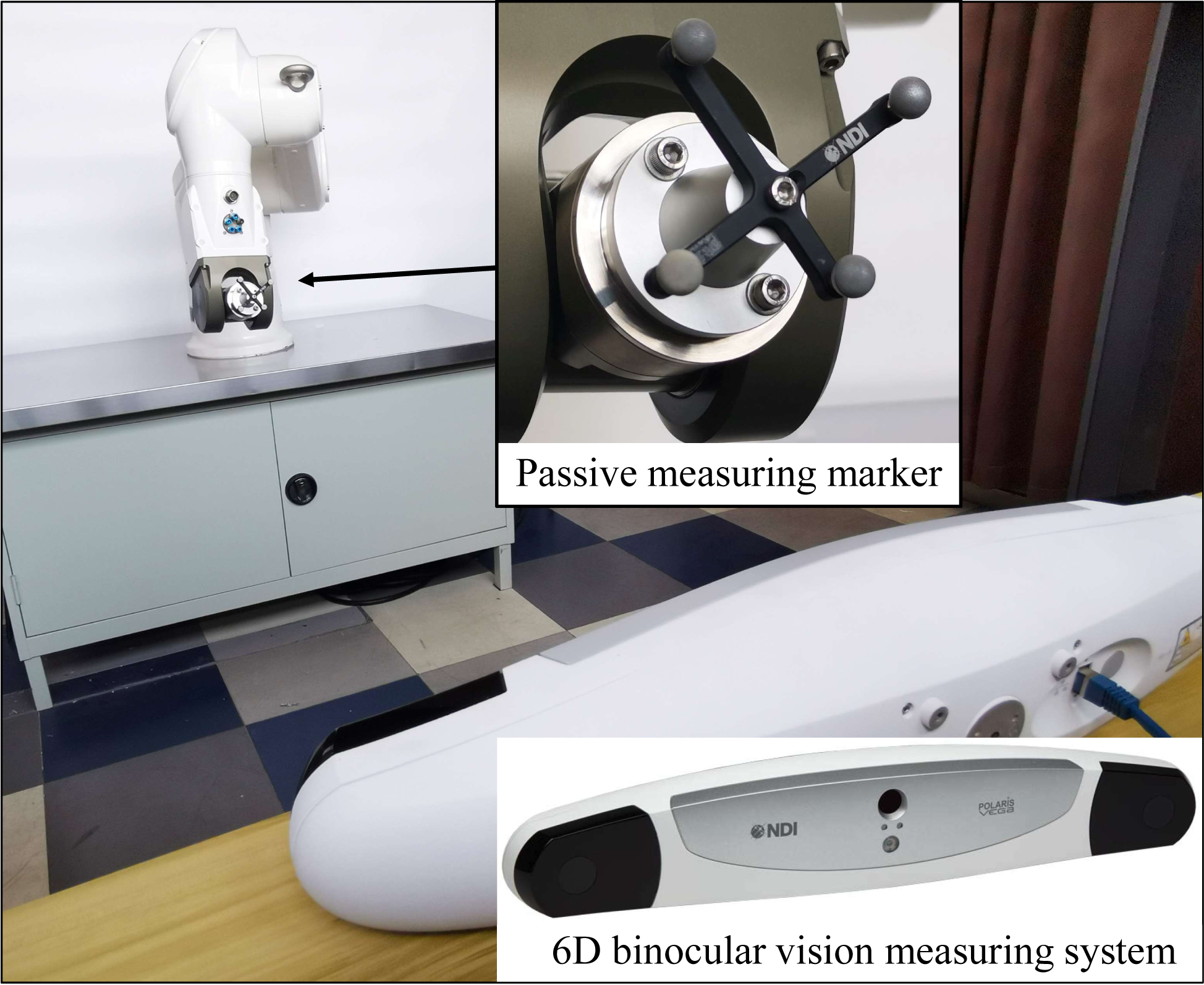

Supplement: Supplementary file 1 [file sensors-23-05717-s001.zip › Figures/Fig9-a.png]

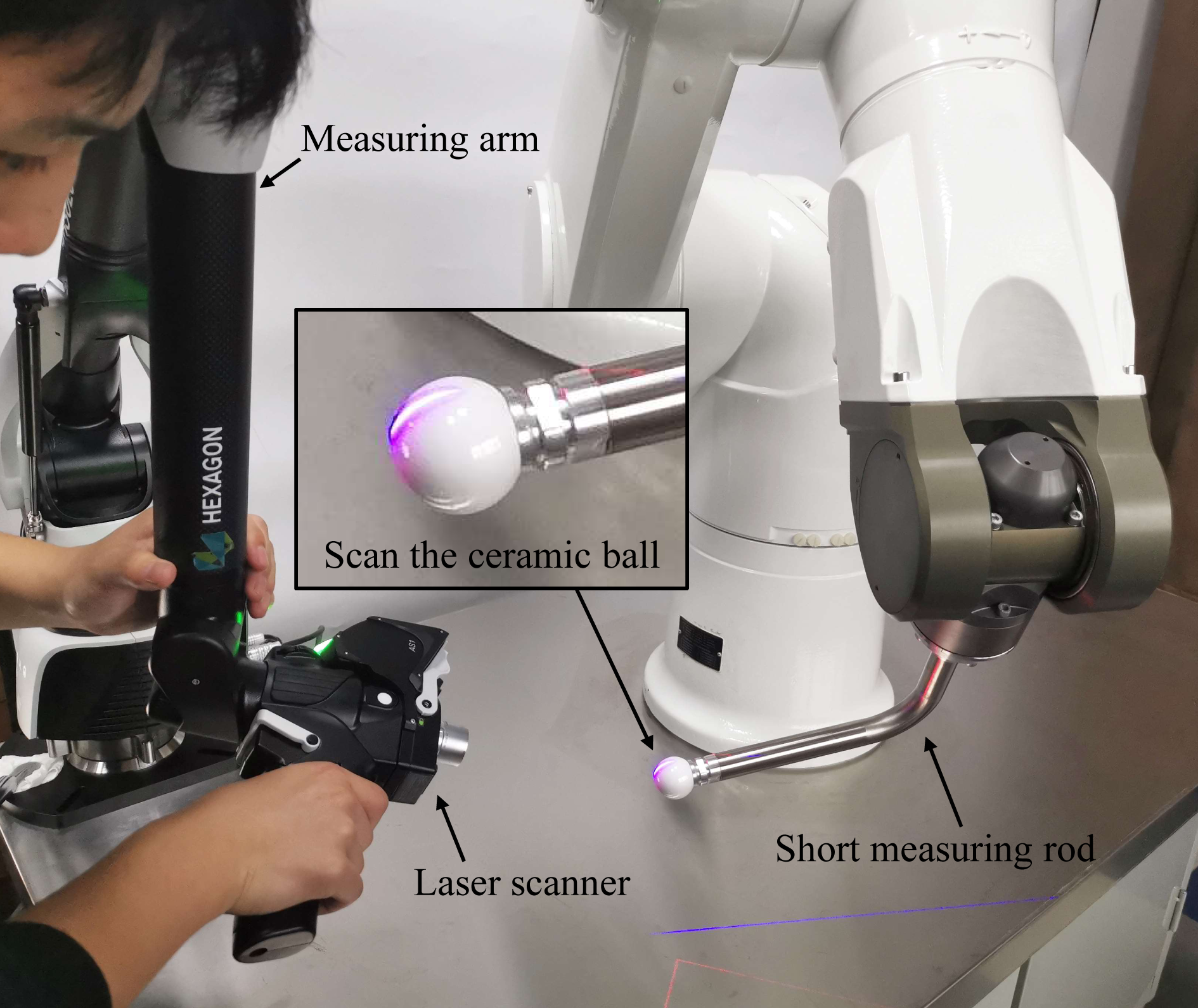

Supplement: Supplementary file 1 [file sensors-23-05717-s001.zip › Figures/Fig9-b.png]

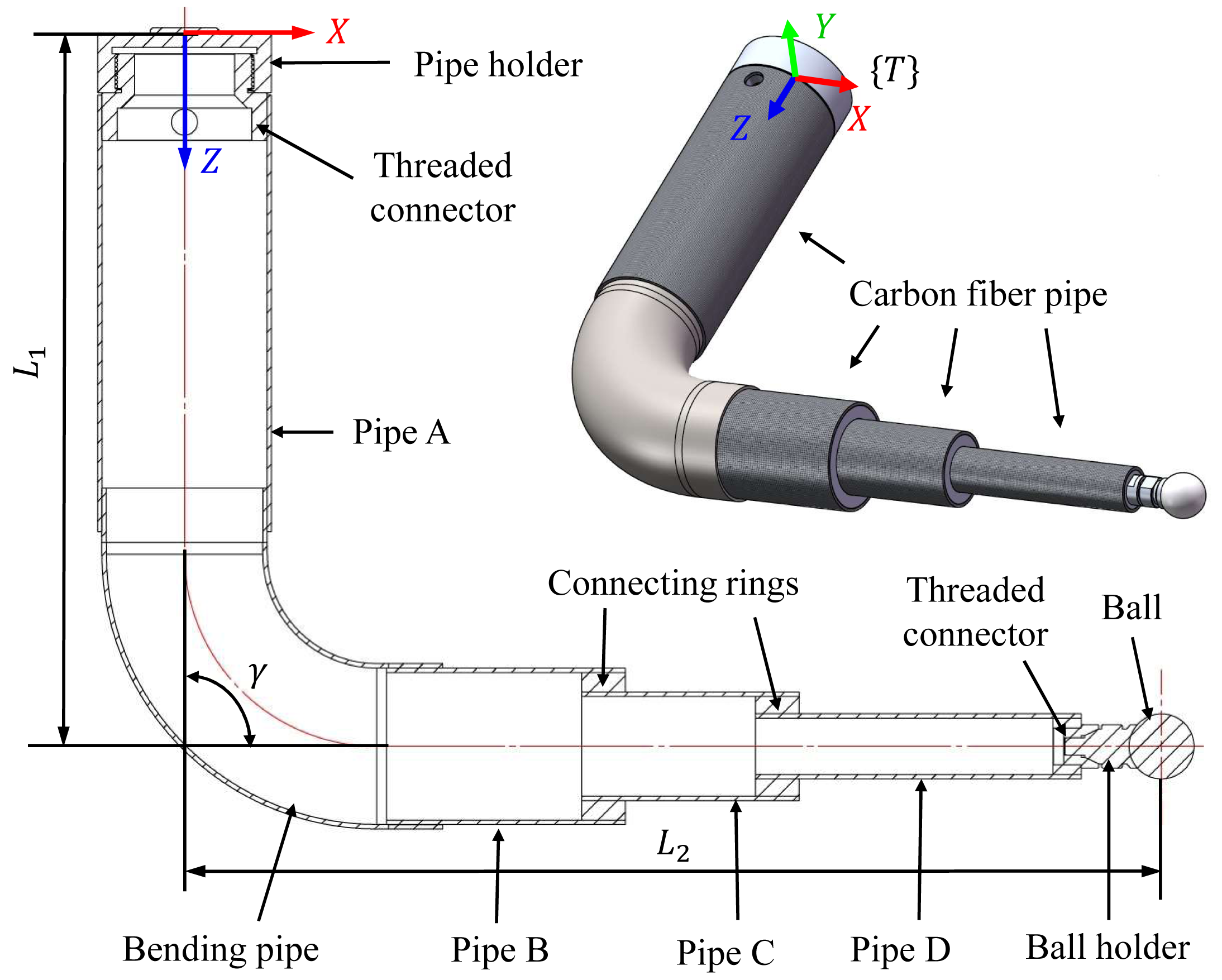

Supplement: Supplementary file 1 [file sensors-23-05717-s001.zip › Figures/FigS1.png]

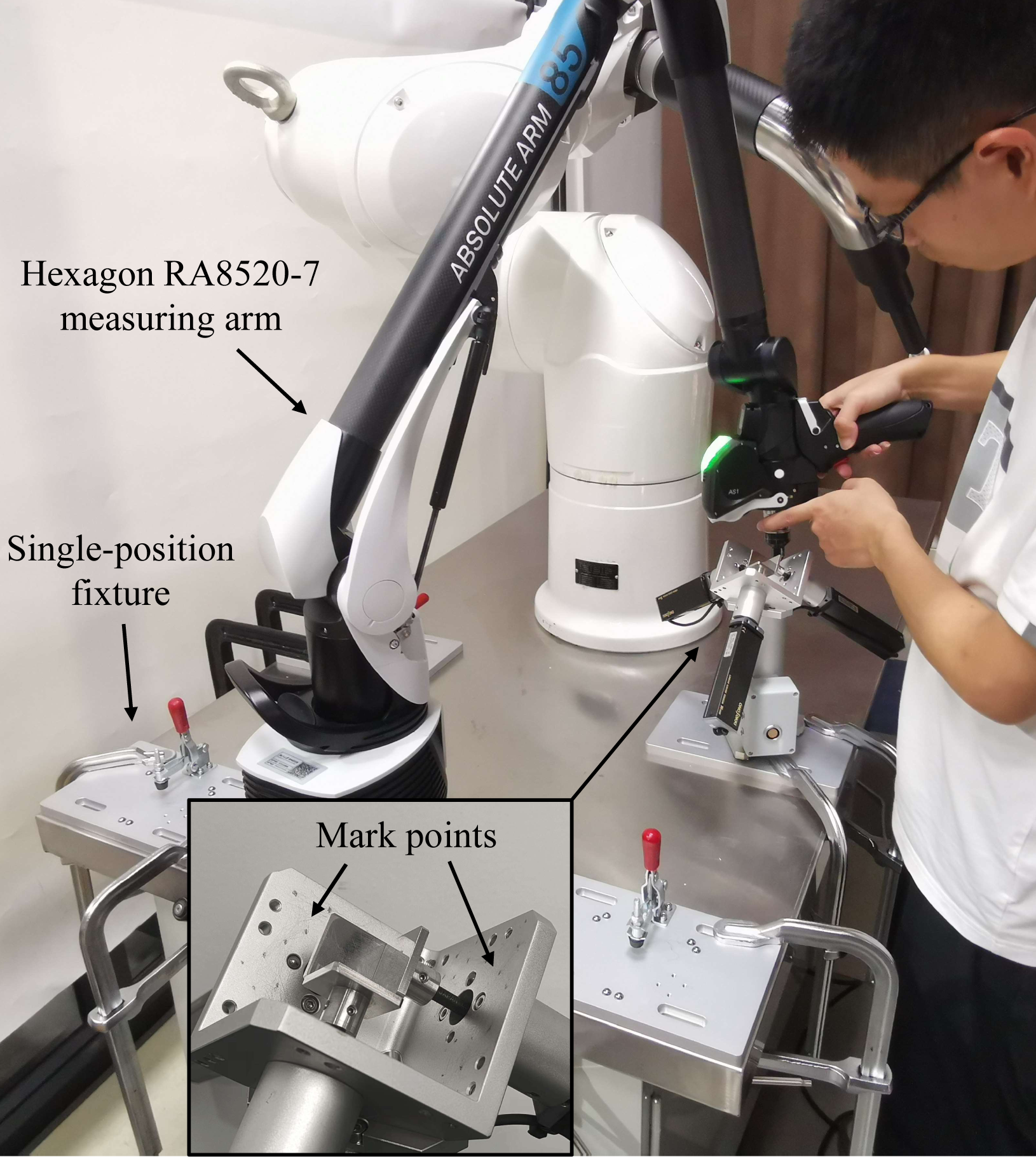

Supplement: Supplementary file 1 [file sensors-23-05717-s001.zip › Figures/FigS2.png]

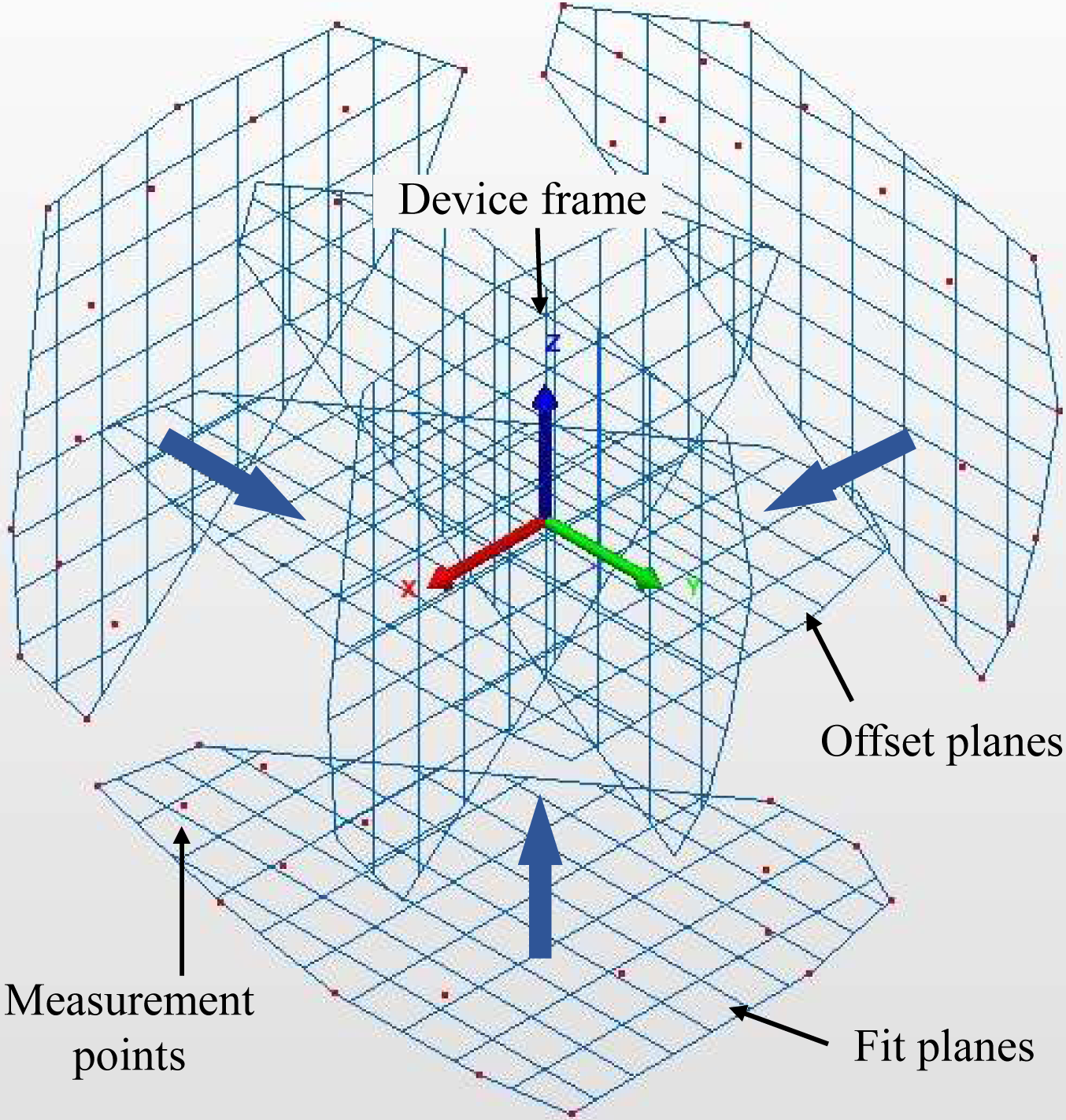

Supplement: Supplementary file 1 [file sensors-23-05717-s001.zip › Figures/FigS3.png]

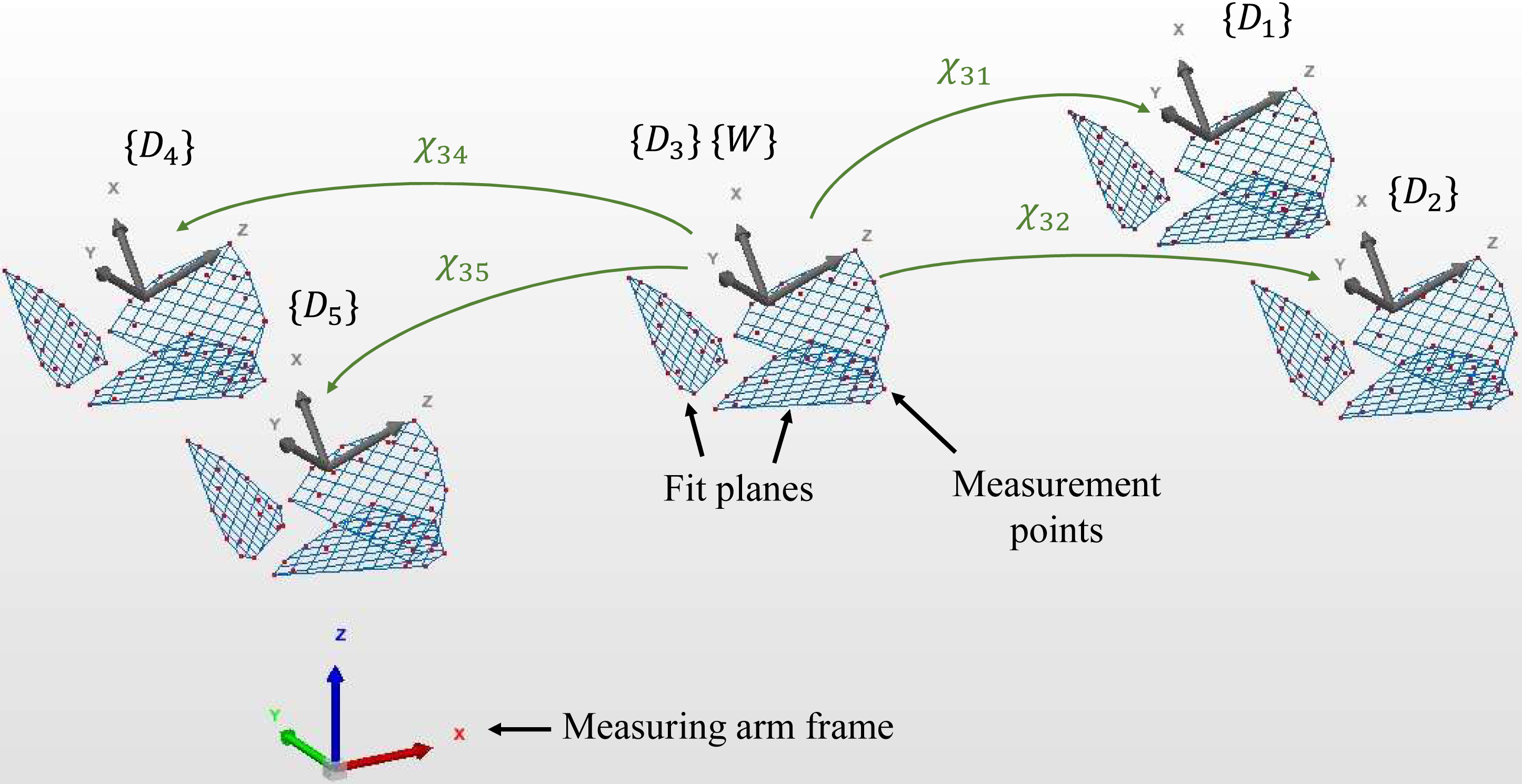

Supplement: Supplementary file 1 [file sensors-23-05717-s001.zip › Figures/FigS4.png]

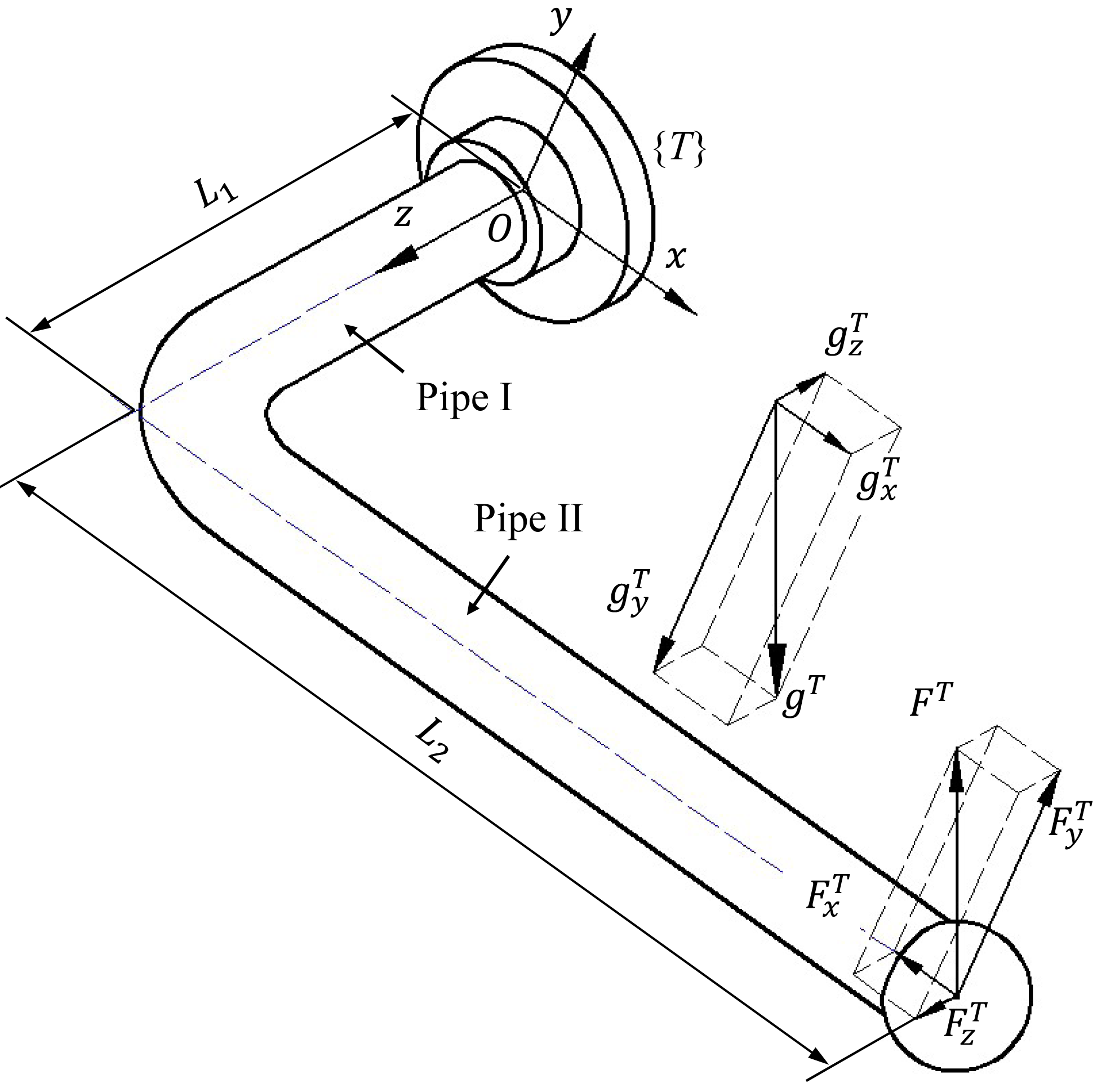

Supplement: Supplementary file 1 [file sensors-23-05717-s001.zip › Figures/FigS5.png]

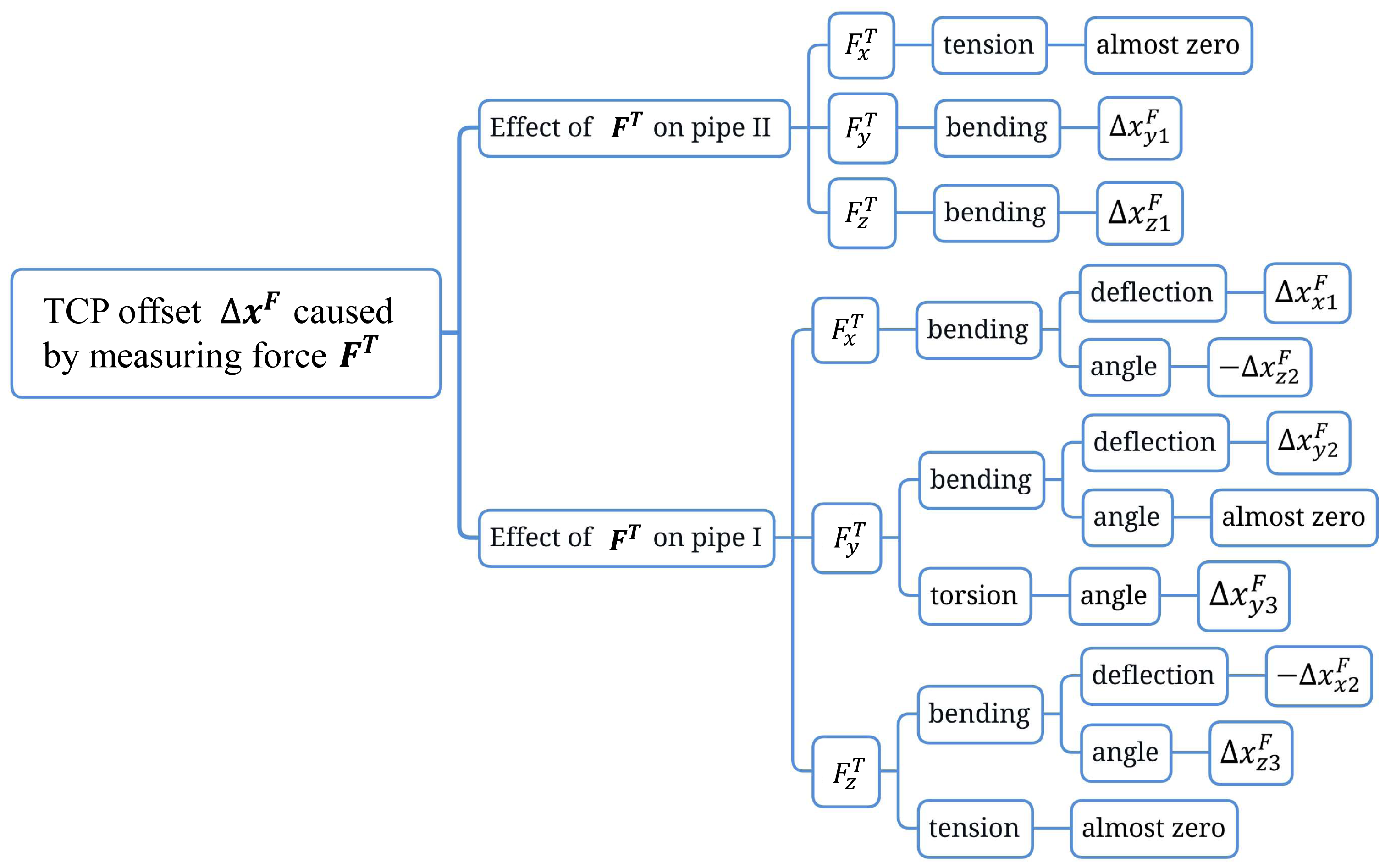

Supplement: Supplementary file 1 [file sensors-23-05717-s001.zip › Figures/FigS6.png]

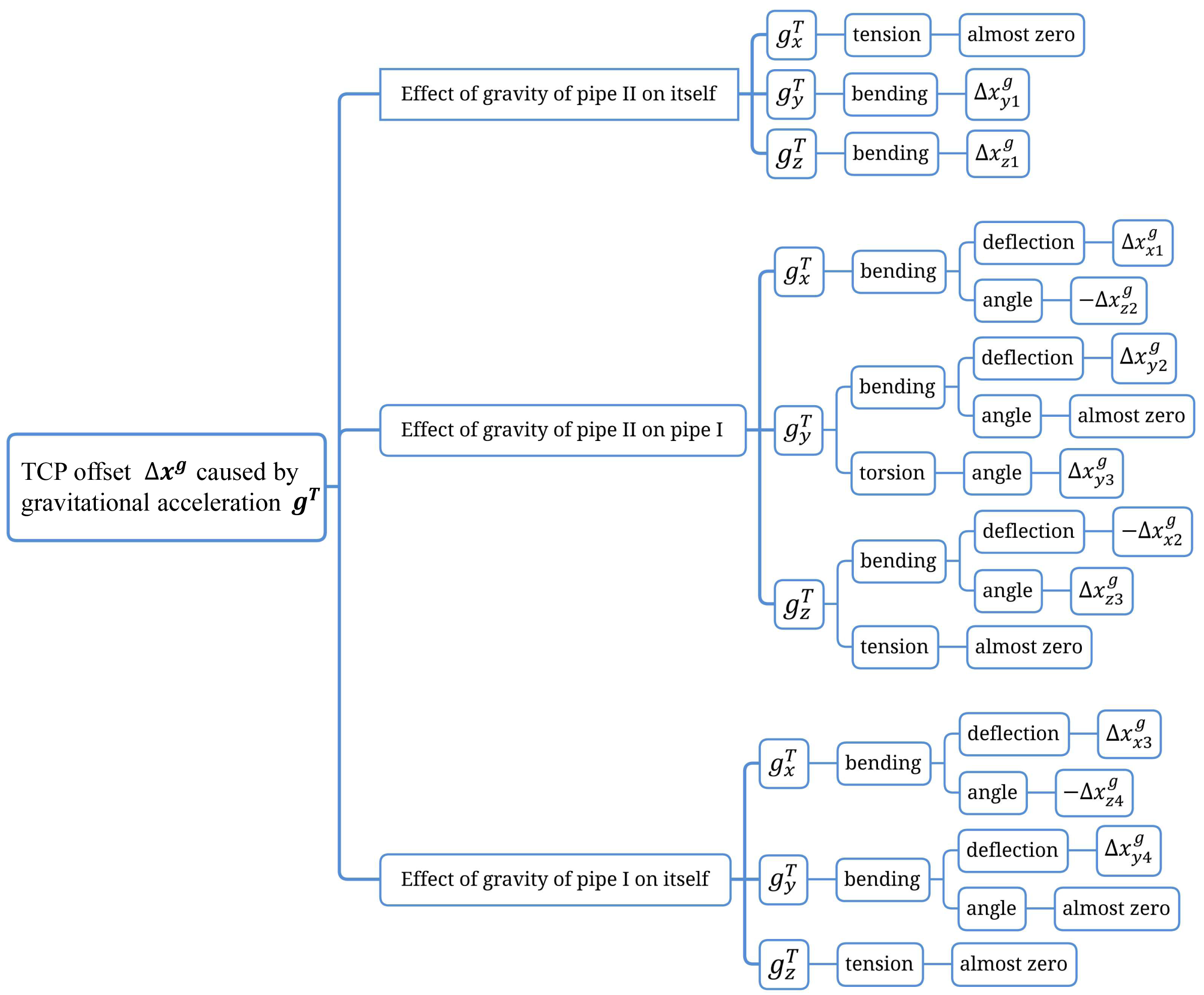

Supplement: Supplementary file 1 [file sensors-23-05717-s001.zip › Figures/FigS7.png]

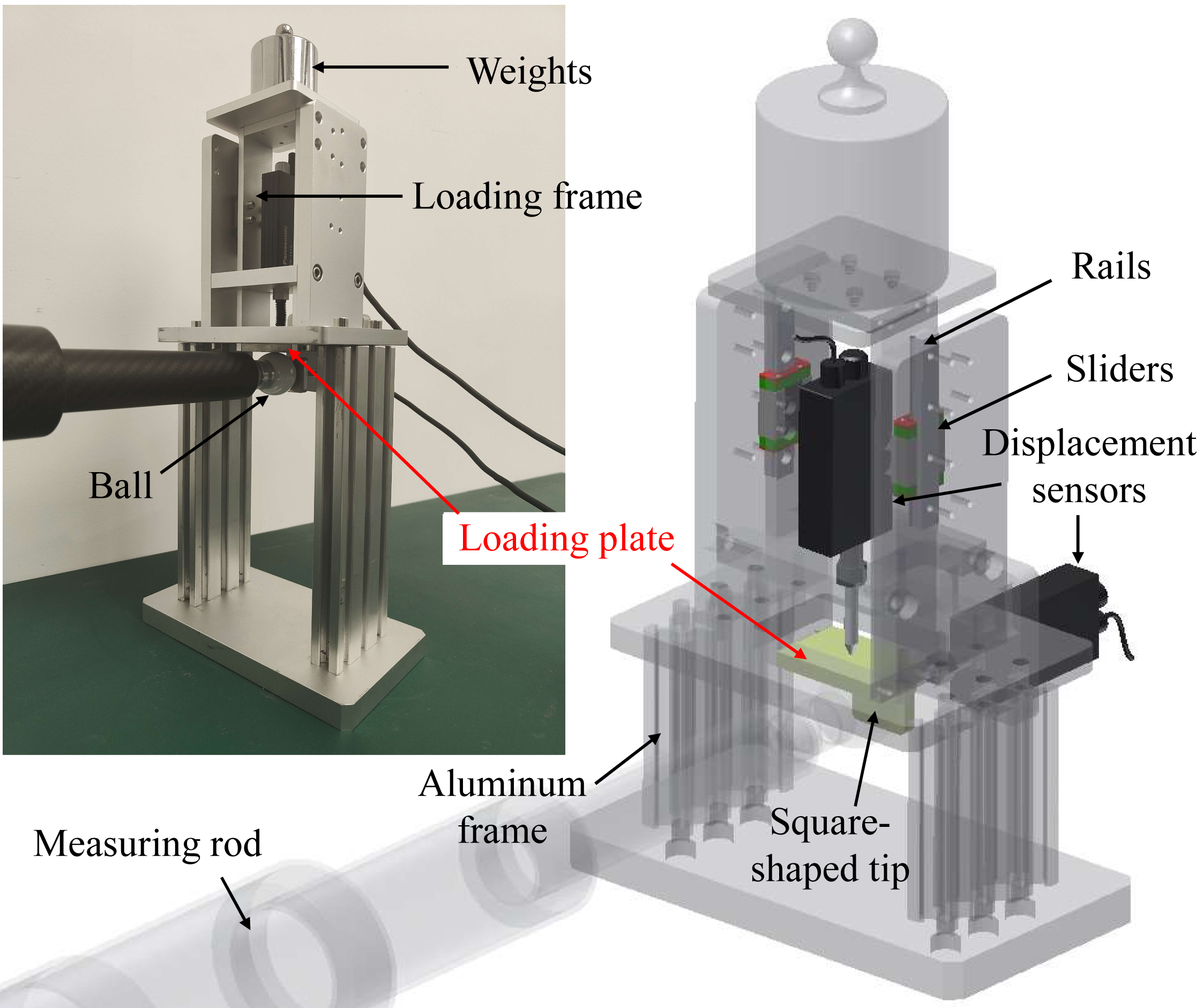

Supplement: Supplementary file 1 [file sensors-23-05717-s001.zip › Figures/FigS8.png]

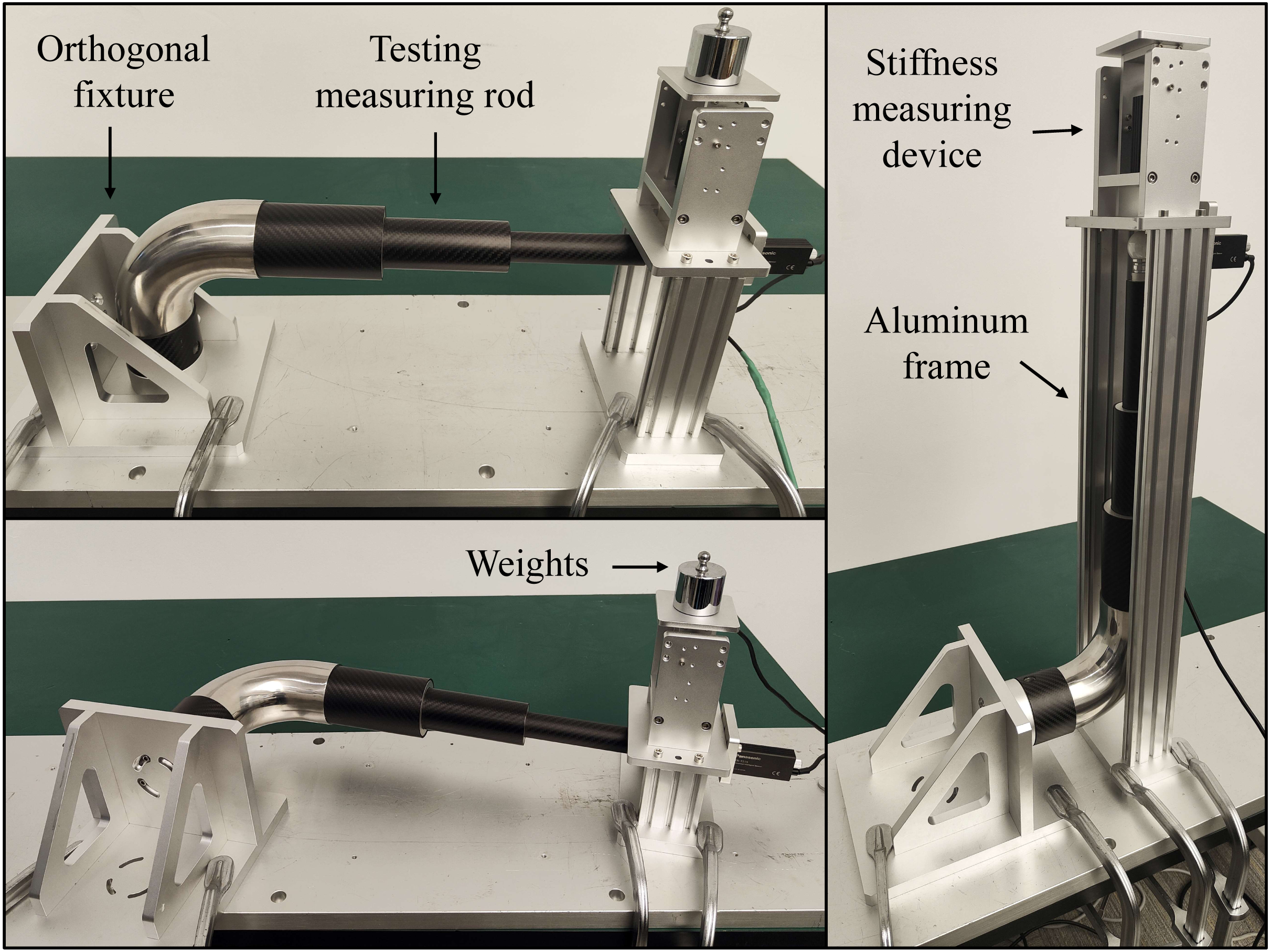

Supplement: Supplementary file 1 [file sensors-23-05717-s001.zip › Figures/FigS9.png]
